# Home use of low-intensity transcranial electrical stimulation in clinical practice: an IFCN handbook chapter

**DOI:** 10.1016/j.cnp.2026.03.006

**Published:** 2026-03-30

**Authors:** Perianen Ramasawmy, Marom Bikson, Jerome Brunelin, Kyle Donnery, Alexander Hunold, Jean-Pascal Lefaucheur, Marine Mondino, Teresa Schuhmann, Antonio Oliviero, Andrea Antal

**Affiliations:** a Non-Invasive Brain Stimulation Lab, Department of Neurology, University Medical Center Göttingen, Göttingen, Germany; b Department of Biomedical Engineering, The City College of New York, NY, United States; c Université Claude Bernard Lyon 1, CNRS, INSERM, Centre de Recherche en Neurosciences de Lyon CRNL U1028, UMR5292, PsyR2 Team, Bron, France; d Le Vinatier Psychiatrie Universitaire Lyon Métropole, 95 boulevard Pinel, Bron, France; e Institute of Biomedical Engineering and Informatics, TU Ilmenau, Ilmenau, Germany; f neuroConn GmbH, Ilmenau, Germany; g UR4391 (ENT Team), Faculty of Health, Paris Est Créteil Université, Créteil, France; h Department of Clinical Neurophysiology, Henri Mondor University Hospital, AP-HP, Créteil, France; i Department of Cognitive Neuroscience, Faculty of Psychology and Neuroscience, Maastricht University, the Netherlands; j FENNSI Group, Hospital Nacional de Parapléjicos, SESCAM, Toledo, Spain; k Instituto de Investigación Sanitaria de Castilla-La Mancha, Toledo, Spain; l Centre for Clinical Neuroscience, Hospital Los Madroños, Brunete (Madrid), Spain

## Abstract

Non-invasive brain stimulation (NIBS) includes a growing set of techniques aimed at modulating brain activity without surgery or implants. Transcranial magnetic (TMS) and electrical stimulation (tES) are among the most established methods. tES delivers low-intensity current via scalp electrodes, offering a cheaper and portable option, especially for home-based use. Clinical evidence suggests that the effects of tES are cumulative with consecutive applications needed to achieve meaningful changes. The therapeutic application of the clinic-based tES usually involves a minimum of two weeks of daily visits to the clinical institute, which poses a large burden and stress on patients. Home-based tES, e.g. under remote supervision (RS-tES), following adequate training by trained professionals paves the path to increasing the accessibility of the technology to patients. In 2025, the US FDA approved the first home-based tDCS system for the treatment of “moderate to severe major depressive disorder in the current episode, either as monotherapy or as an adjunctive treatment, in patients 18 years and older who are not considered treatment refractory to medication. In this work, the latest knowledge related to home-use of tES is introduced, including the methodology, most frequent clinical applications, advances and limitations.

## Highlights

• tES delivers low-intensity current via scalp electrodes, offering a portable option.
• Home-based tES paves the path to increasing the accessibility of the technology.
• Providing tES treatment at home improves compliance.

## 1 Introduction

Non-invasive brain stimulation (NIBS) refers to an expanding array of techniques designed to modulate neural activity in a non-invasive manner (e.g., without the need for surgical intervention or implanted devices). Alongside their development as tools for investigating brain function, these methods have evolved into therapeutic modalities with growing clinical relevance. Today, NIBS is employed in the treatment of a wide spectrum of psychiatric and neurological conditions, including major depressive disorder, stroke, chronic pain, and cognitive dysfunctions (Lefaucheur et al., 2017, Lefaucheur et al., 2020, Lisanby, 2024). Among the most well-established NIBS techniques are repetitive transcranial magnetic stimulation (rTMS) and transcranial electrical stimulation (tES), both of which have substantial empirical and regulatory support (Antal et al., 2017, Antal et al., 2025a, Desarkar et al., 2024). rTMS uses pulsed magnetic fields to induce electric currents in cortical tissue and was first approved by the U.S. Food and Drug Administration (FDA) for treatment-resistant depression (Janicak et al., 2008, Lisanby et al., 2009, O’Reardon et al., 2007). Its clinical use is mainly restricted by the need for high-cost equipment, and by the necessity to be applied in specialized clinics and by specially trained personnel. However, the ongoing development of portable light-weight rTMS devices for home use (Qi et al., 2025) represents a promising avenue for enhancing both the accessibility and scalability of the technology.

In contrast, transcranial direct current stimulation (tDCS), the most widely used form of tES, operates by delivering low-amplitude direct current (typically 1–2 mA) through electrodes placed on the scalp (Nitsche and Paulus, 2000). In contrast to TMS, which induces action potentials in cortical neurons (Klomjai et al., 2015), tDCS works by sub-threshold modulation of the resting membrane potential, thereby altering the probability of neuronal firing (Nitsche and Paulus, 2000, Stagg et al., 2018). Beyond the local effects of tDCS, the network effects and potential long-term changes in cortical excitability through repeated stimulation forms the basis for its therapeutic benefits (Lefaucheur et al., 2017, Stagg et al., 2018, Woods et al., 2016). Its relative simplicity, affordability, and favorable safety profile make it particularly suited for increasing the availability of NIBS even as a home-based application (Charvet et al., 2015, Woodham et al., 2025a). Conversely, transcranial alternating current stimulation (tACS)—another tES technique—applies sinusoidal currents to modulate activity in specific brain regions Offline effects arise from neural entrainment and neuroplastic changes (Elyamany et al., 2021). Although tACS shows exponentially growing potential for clinical use, most evidence on home-based tES focuses on tDCS.

tDCS is typically applied through two large (25–100 cm^2^) rubber electrodes, applied either with conductive cream or within saline-soaked sponges to direct current delivery. Regarding terminology, we follow conventional practices including: 1) using “anodal” or “cathodal” tDCS to indicate focus in the anode or cathode electrodes, respectively, without implying that only one electrode is active; 2) a brain region is “targeted” (e.g., “prefrontal or precentral-tDCS”) in the limited sense that an electrode is placed over the region, without implying that the current is delivered focally (only) to that region; 3) duration including ramp on/off; 4) intensity is reported as peak for tDCS and as peak-to-peak amplitude for tACS. Conventional low-intensity tES (Antal et al., 2017, Bikson et al., 2019), also known as limited-output tES (Bikson et al., 2019), generally refers to peak currents at or below 4 mA. This does not suggest that modestly higher currents—such as 6 mA (Bikson et al., 2016, Donnery et al., 2025)—are not tolerated or unsafe, however, using higher intensities, limited data are available.

Evidence from both basic research and clinical studies suggest that the effects of tDCS can be cumulative, such that multiple applications are needed to achieve clinically meaningful benefits. This implies that the stimulation must be repeated daily or even several times during a day (accelerated protocols). A challenge in the clinic is how to complete the necessary number of treatments. Providing tDCS treatment outside the clinic, e,g, at home, can decrease burdens for patients and their caregivers by decreasing the number of days when they need to travel to the medical facilities. Other advantages include increased treatment compliance and access to tDCS for patients who live in geographically remote areas or live with physical or cognitive disabilities. The possibility of home-based tES has been applauded by different stakeholder groups, including individuals with lived experience and industry representatives (Antal et al., 2024, Maier et al., 2024, Ramasawmy et al., 2024).

Home-based tDCS technologies have significantly evolved and are increasingly recognized for their therapeutic potential across diverse clinical applications. Clinical research supports the efficacy of home-based NIBS in managing symptoms, for example, of depression or pain (Bréchet et al., 2021, Charvet et al., 2015, Vogelmann and Baskonus, 2025, Vogelmann et al., 2025, Woodham et al., 2025b). Improvements in home-based tDCS technologies are directed toward ensuring tolerability, user-friendliness, and facilitating real-time remote supervision. With the rapid expansion of telemedicine, these devices designed for home use are increasingly integrated with tele-supervised frameworks, enabling healthcare providers to remotely monitor patients and adjust stimulation parameters as necessary (Chirra et al., 2019, DaSilva et al., 2022, Riggs et al., 2018). Research evidence furthermore strongly supports the combination of home-based NIBS with adjunctive therapies such as cognitive training, physical rehabilitation exercises, stress management, and mindfulness-based interventions. Indeed, multimodal therapeutic strategies harnessing the complementary strengths of NIBS and behavioral interventions have been shown to amplify therapeutic effects significantly (Cavendish et al., 2022, Neige et al., 2024, Wickens et al., 2025).

Direct-to-consumer tDCS devices for neuroenhancement have expanded over the past years (Bourzac, 2016, Jwa, 2015) which has led to discussions surrounding the safety, ethical, and societal consequences of non-therapeutic home-based tES. Beyond the clinical applications of tDCS, its implementation for military use (Sehm and Ragert, 2013), use in sports (Pugh and Pugh, 2021), and neuroenhancement in healthy individuals (Brukamp et al., 2012) require special considerations.

## 2 Desired characteristics of NIBS devices that can be used at home

For any medical intervention, there are associated risks that govern the degree of professional oversight and controls warranted. tES, including tDCS, has been extensively studied in academic and medical centers, demonstrating its safety and tolerability (Antal et al., 2017, Bikson et al., 2016). Since low-intensity tES devices are relatively low-cost, battery-operated, with straightforward operation and diverse applications, there is an interest and a potential for home use. However, the translation of tES from academic or medical centers to home use requires careful consideration of both technology and protocols to ensure a reproducible set-up. Most of the brain stimulation devices and accessories designed for use by trained professionals in clinical or research settings are generally not suitable for home use. For example, devices designed for academic/medical centers may allow for a wide range of doses and unlimited repeated activation, whereas home-based devices should provide a specific prescribed and limited dose. Stimulation devices designed for home use may be modified to provide reduced voltage (Bikson et al., 2018, Hahn et al., 2013), with single position headgear (DaSilva et al., 2022, Knotkova et al., 2019), single-use and/or pre-prepared electrodes (Borges et al., 2020), or caps with integrated electrodes (Hunold et al., 2020), simplified impedance testing and controls, as well as methods for remote device deactivation. Home-based tES can be combined with a range of mobile-health or digital healthcare technologies (Brunoni et al., 2022), but protocols for reproducible stimulation still need to be ensured.

Specifically, the safety and tolerability observed in academic or medical settings only apply to home use if the relevant stimulation protocols are faithfully reproduced and devices meet appropriate standards. This includes ensuring that the stimulation parameters—such as intensity, duration, and frequency—remain within clinically validated (safe) ranges. The device should have built-in safeguards to prevent misuse, such as automatic shutoff features, current limiters, and continuous monitoring for skin contact quality or impedance. It should also be designed to minimize side effects and adverse events like skin irritation or discomfort, thereby ensuring tolerability over repeated sessions.

Ideally, the device must be user-friendly and suitable for individuals without medical or technical backgrounds. It should feature an intuitive interface with simple instructions, automated calibration, and pre-programmed stimulation protocols (Pilloni et al., 2021, Zhong et al., 2024). Visual or auditory cues can help guide users through proper setup and indicate when stimulation is active or complete. The design should minimise the chance of human error during setup.

For a device to be well suited for home-based use, it needs to be lightweight, compact, and ergonomically designed. The hardware—whether configured as a headset or cap—must ensure user comfort during prolonged wear, minimizing physical strain or slippage. Wireless connectivity or battery-powered operation enhances user mobility and allows stimulation in different home settings, such as while sitting, lying down, or even performing light tasks. The ability to pack and store the device easily also contributes to long-term adherence.

From the operator/clinical side, the device should offer flexible settings to accommodate individual treatment plans and evolving therapeutic needs. Whether used for depression, chronic pain, or cognitive enhancement, personalization is critical. While the devices require fixed stimulation protocols for the patients to ensure safety, they need to allow clinicians to update settings remotely based on patient progress. Features like adaptive stimulation (responding to biofeedback such as EEG signals) or multi-site targeting are not common in home-based tES but might enhance therapeutic impact. Systems may benefit from a support cloud-based data logging, enabling clinicians to track session adherence, stimulation parameters, and patient-reported outcomes either in real time or retrospectively. Additionally, many platforms benefit from telehealth integration, allowing for live video consultations, remote troubleshooting, and personalized protocol adjustments.

To support monitoring and safety, a wide range of ancillary devices can be integrated with home-based NIBS platforms. These include wearable EEG headsets for monitoring brain activity and verifying target engagement, as well as skin impedance sensors that ensure proper electrode contact and reduce the risk of ineffective stimulation. In the case of tES, this latter feature is an essential safety measure which needs to be incorporated in the device anyway. Inertial sensors and accelerometers can monitor posture, movement, and head position to confirm proper device placement during sessions. Some systems may employ smart cameras or augmented reality (AR) guidance tools for electrode positioning, using image recognition algorithms to provide real-time feedback to users.

Additionally, mobile applications can serve as interactive user interfaces, guiding session setup, collecting symptom ratings, issuing reminders, and delivering progress reports. Integration with wearable physiological trackers (e.g., heart rate monitors, galvanic skin response sensors, sleep trackers) allows the system to monitor broader health parameters and adapt protocols based on stress, fatigue, or sleep quality.

Beyond monitoring, home-based NIBS systems can be significantly enhanced by integrating devices for motor and cognitive rehabilitation, facilitating synergistic, multimodal interventions. The efficacy of neuro-psychiatric interventions can be monitored by e.g., questionnaire-based symptom rating. For motor rehabilitation, this includes tablet-based or VR-enabled physiotherapy apps, robotic gloves, exoskeletons, electromyography (EMG) feedback systems, and motion-capture cameras. These tools allow patients to engage in repetitive, task-specific training aligned with stimulation sessions, which is crucial for neurorehabilitation uses.

For cognitive rehabilitation, a range of digital cognitive training platforms, gamified brain-training apps, and neurofeedback tools can be used in conjunction with NIBS. These platforms often include tasks targeting attention, memory, executive function, or language processing, and can be tailored to specific patient profiles. Some systems use adaptive difficulty algorithms that adjust the cognitive load in real time, maximizing engagement and plasticity. When synchronized with stimulation timing (e.g., during or immediately after NIBS), these interventions may enhance neuroplastic effects and functional gains.

Cost is a factor influencing the widespread adoption of home-based NIBS. The ideal device should be affordable not only for healthcare systems, but also for individuals paying out-of-pocket. This includes keeping production costs low, and offering options for rent or insurance coverage. Devices should also be accessible for users with disabilities or mobility issues.

Finally, the device must comply with medical regulatory standards, such as CE marking in Europe or FDA clearance in the U.S. This ensures that its use is supported by evidence from clinical trials demonstrating safety, efficacy, and usability in home environments. Evidence-based design also fosters trust among clinicians and patients alike. Clear instructions for use, training materials, and customer support should accompany the device, promoting safe, informed, and consistent use over time.

### 2.1 Home stimulation devices: monitoring and guidelines

One of the most important points when home stimulation is applied, the adequate patient and/or caregiver training by expert healthcare professionals. Unlike procedures in controlled clinical environments, home use introduces variability in device handling, setup and session timing. Key risks include improper device preparation, electrode placement, and lack of recognition of adverse events such as headaches, skin irritation, or transient cognitive changes. To mitigate these risks, home-based systems must incorporate resilience to handling errors, automated safety mechanisms, including stimulation limits, guided setup instructions, impedance checks, and real-time feedback to ensure correct use. Not all patients are suitable candidates for unsupervised home-based stimulation. Individuals with severe cognitive impairment, active psychiatric disorders, or poor treatment insight may struggle to use these devices safely and consistently. Therefore, appropriate patient selection criteria are essential, along with structured education and training for both patients and caregivers. In some cases, home-based NIBS should be restricted to those with prior in-clinic experience or integrated into hybrid care models that combine remote monitoring with periodic clinical check-ins to balance safety with autonomy.

The second critical issue is the supervision and monitoring of the use of the stimulator and stimulation sessions at home by the clinical team. Varying approaches and different levels of supervision and monitoring to at-home use of tDCS have been described in the literature to date (Abdullahi et al., 2024, DaSilva et al., 2022, Moshfeghinia et al., 2025). Many home stimulator devices can be used in a “supervised” mode, in which the traceability of the sessions and monitoring of their effective implementation (e.g. time, impedance, number of sessions). The most rigorous approaches to at-home tDCS include the application of comprehensive clinical protocols with high methodological control that includes real time monitoring of the sessions performed by the patient at home by an operator at the hospital via videoconference (at least for some of the treatment sessions) so that correct headset placement can be ensured. This type of approach is obviously the best way to guarantee the correct application and its safety.

For example, a protocol termed “Remotely Supervised” or RS-tDCS, uses standardized procedures with ongoing supervision during treatment with patient-tailored at-home tDCS. For this procedure a guideline was developed, based on eight items that inform the reproducible applications of limited-output tES under supervision of a medical professional or researcher (Charvet et al., 2015). The recommendations include: (1) comprehensive training of individuals administering and supervising the tDCS; (2) evaluation of each user’s ability to safely engage in remote tDCS; (3) provision of continuous training materials and competency assessments for users and caregivers; (4) implementation of simple, fail-safe electrode placement methods and standardized headgear; (5) enforcement of strict dose control across all sessions; (6) real-time monitoring of compliance parameters, with corrective actions as needed; (7) systematic observation and documentation of any treatment-emergent adverse events; and (8) clearly defined procedures for terminating individual sessions or study participation, including tailored emergency failsafe protocols appropriate to the treatment population. RS-tES provides significant flexibility for diverse applications (for example, the degree of video supervision needed: RS-tDCS does not require ongoing telemedicine, but it explicitly allows each trial to decide on the appropriate level of supervision) under a rational rubric of rules (Charvet et al., 2020). RS-tES, especially RS-tDCS, has been broadly applied with reliable success in diverse patient populations (Agarwal et al., 2018, Pilloni et al., 2022, Shaw et al., 2020, Simpson et al., 2022).

If a home-based tES protocol is run without ensuring and documenting reliability, the outcomes of that experience (whether positive or negative) cannot be re-applied or generalized. RS-tES does require initial and ongoing supervision by healthcare professionals and researchers. Self-directed tES (e.g. direct to consumer) is not RS-tES.

The importance of using proper equipment and protocols in tES is clear: e.g. burns (stimulation-induced skin lesions) do not occur when established best practices are followed (Woods et al., 2016), but have been reported when these practices are not adhered to (Pilloni et al., 2021). This dichotomy also extends to home-based tES. Burns are not expected in RS-tDCS (Pilloni et al., 2022), but can occur in non-RS-tDCS home-based approaches, along with an increased incidence of side effects and adverse events (Vogelmann and Baskonus, 2025, Vogelmann et al., 2025).

## 3 Home-based tES in psychiatric disorders

Psychiatric disorders represent a major public health concern, with approximately 13% of the world’s population living with a psychiatric condition. Among them, about one in three patients shows only partial or limited responses to conventional treatments. In this context, particularly tDCS, has attracted growing interest as a well-tolerated, non-invasive, and complementary therapeutic approach. Recent advances in portable and user-friendly devices have made it possible to deliver tES at home under remote clinical supervision (RS-tES). Several studies conducted over the past few years suggest that home-based tDCS could be a promising therapeutic strategy for psychiatric conditions, while maintaining a high level of safety through remote monitoring by clinical teams. While home-based tES has been studied across different psychiatric and neurological disorders, the significant heterogeneity in the stimulation parameters across studies such as current intensity, duration of stimulation, variable electrode montage emphasizes the current lack of standardized home-based protocols for widespread clinical prescription. The evidence of safety and clinical efficacy as well as the stimulation protocols of the studies cited in this section is summarized in Table 1.

**Table 1:** Summary of studies cited in section on Home-based tES in Psychiatric Disorders.

#	Author	N	Study design	Training session	Administration /monitoring	tES type	Electrode position	Intensity (mA)	Duration	# of sessions	Sham protocol	Major finding	Side effect (SE)/adverse event (AE)
Major Depressive Disorder													
1	Alonzo et al. 2019	34	Open-label trial	On-site training and assessment of training via checklist of procedures.	Self-administered, Monitored via video link for first 3 sessions by research staff, then video link i as needed. Also an online treatment diary.	tDCS	Anode-F3; Cathode- F8	2	30 min	Group 1: 20 sessions once per day over 4 weeks Group 2: 32 sessions (acute phase: once daily over 4 weeks and taper phase: 4 tDCS sessions spaced 1 week apart)	—	Significant improvements in mood up to 1 month post-acute phase.	SEs were largely transient and minor. Most commonly reported SE were mild to moderate tingling or burning/heat sensation during stimulation and skin redness under electrodes. Comparable to clinic-based studies.
2	Kumpf et al., 2023	11 (5 active)	RCT	On-site supervised training session.	Self-administered, safety monitoring by calling study team in case of presence of AEs.	tDCS	Anode-F3; Cathode- F4	2	30 min	30 sessions (5 sessions per week for 6 weeks)	Same ramp-in (15 s) and ramp-out (15 s) but without intermittent stimulation.	Significant reduction in depression scales over time, without any group difference between sham and real tDCS.	Study had to be stopped due to 5 AEs (skin lesions).
3	Cappon et al., 2021 Older adults with MDD	5	Case series (2 patients withdrew due to medical conditions unrelated to study treatment)	Home-based training for study companion via training sessions via video-conference, self-directed learning via video and paper-based material.	Companion-administered, remote monitoring of progression during each session via a smart tablet. On demand remote assistance available.	Multichannel tDCS	Anode: F3; Cathodes: FZ, FC5, FP1	Max current per electrode around 1.75 mA	30 min	37 sessions (acute phase: once daily over 4 weeks and taper phase: 9 tDCS sessions over 4 weeks [first 3 every second day, next 3 every third day, and last 3 every fourth day)	—	Beneficial effects on depression symptoms comparing baseline to one-month follow-up.	SEs were mild and transient. Most frequently reported SEs were sensations under the electrodes such as tingling and itching, post-stimulation sleepiness, scalp redness, and neck pain.
4	Ruffini et al., 2024	35	Open-label multicentre; combined with app-based behavioural therapy	Home-based training for study companion or participant via training sessions via video-conference, self-directed learning via video and paper-based material.	Self/companion-administered, remote monitoring of progression during each session via a smart tablet. On demand remote assistance available.	Multichannel tDCS	Anodes: F3, AF3; Cathodes: T7, AF4	3.1 (total injected current in group-optimized montage)	30 min	37 sessions (acute phase: once daily over 4 weeks and taper phase: 9 tDCS sessions over 4 weeks [first 3 every second day, next 3 every third day, and last 3 every fourth day)	—	Median reduction in depressive symptoms 4 weeks post-treatment with responder rate of 72.7%.	No Serious AEs were observed. No participants showed suicidal ideation/behaviour.
5	Sobral et al 2022	7	Case series	Training by a clinical psychologist.	Self-administered tDCS, weekly appointment with psychologist for monitoring.	tDCS (combined with Flow™ app-based behavioural therapy)	Anode-F3; Cathode- F4	2	30 min	Protocol 1: 5 sessions per week (once per day) for 2 weeks, followed by twice-weekly 7sessions for 4 weeks (n = 18) Protocol 2: 5 sessions per week (once per day) for 3 weeks, followed by twice-weekly sessions for 3 weeks (n = 21)	—	Clinically meaning reduction in depressive symptoms in 5 patients on the MADRS-S and in 4 patients on the BDI-II.	Well-tolerated, without any severe SEs. Most frequent AEs were scalp irritation, tingling, itching, and burning sensation.
6	Borrione et al., 2021	5	Case series	Supervised training with specialized staff.	Unsupervised self-administered tDCS, remote access to staff in case of questions or complications.	tDCS (combined with Flow™ app-based behavioural therapy)	Anode-F3; Cathode- F4	2	30 min	21 sessions (5 sessions per week (once per weekday) for 3 weeks, followed by twice-weekly sessions for 3 weeks)	—	Three treatment responders on the HDRS, 3 of which went in remission. Four patients showed substantial improvement in BDI-II and MADRS scores.	No serious AEs or complications.
7	Borrione et al., 2024	210	RCT	Supervised training with specialized staff.	Unsupervised self-administered tDCS.	tDCS (combined with Flow™ app-based behavioural therapy)	Anode-F3; Cathode- F4	2	30 min	21 sessions (5 sessions per week (once per weekday) for 3 weeks, followed by twice-weekly sessions for 3 weeks)	Same as active but current active at the initial and last 45 s of each session and max intensity of 1 mA.	No statistical difference in depressive symptoms among active tDCS combined with digital behavioural intervention (double active), sham tDCS paired with digital placebo [free internet browsing] (double sham) and active tDCS combined with digital placebo (tDCS only).	No group differences in frequency, number, and severity of AEs. Local skin redness commonly reported in double active and tDCS only groups. Burning sensation more commonly reported in double active than double sham group.
8	Koutsomitros et al., 2023	40	Open-label	Supervised training by certified tES practitioner.	Self-administered tDCS, remote monitoring of data within 24-hour timeframe each day by a trained clinician.	tDCS combined with psychotherapy (n = 20) versus psychotherapy only (TAU; n = 20)	Anode-F3; Cathode- F4	2	30	21 sessions (once per day over 3 weeks)	—	Greater reduction in depressive symptoms, greater treatment response and remission rates in tDCS group compared to TAU group.	Mild to moderate discomfort such as slight headache commonly reported. Most severe SEs were two instances of scalp pain. No serious AEs were reported.
9	Woodham et al., 2025a, Woodham et al., 2025b	174	RCT	First session conducted under supervision of trained researcher.	Self-administered tDCS, remote monitoring with real-time data use.	tDCS	Anode-F3; Cathode- F4	2	30	5 sessions (once per day) for 3 weeks and thrice-weekly for 7 weeks (n = 36)	Initial ramp up from 0 to 1 mA over 30 s then ramp down to 0 mA over 15 s. Same applied at end of session.	Reduction in depressive symptoms measured by HDRS in active versus sham group.	At the end of treatment, reports of skin irritation, trouble concentrating, and skin redness were more frequent in active than sham group. No group difference in other SEs and AEs such as headache, neck pain, scalp pain, itching, burning sensation, sleepiness or acute mood changes. Two patients in active group described ‘burns’ at the anode: no lesion or scarring were present. No serious AEs reported.
10	Gehrman et al., 2024	255	RCT	No information provided.	Self-administered; no information provided.	pulsed tACS	The squamous temporal bone above the posterior aspect of the zygomatic arch on either side of the head,	2	20	2 daily sessions for 4 weeks	Same montage but did not deliver electrical stimulation.	Intent-to-treat analysis showed no significant difference between groups. Significant improvements in the active vs sham group emerged in participants reporting twice-daily use every day in the first two weeks.	Low AE rate. 19 participants in active group reported 34 events an 10 in sham group reported 13 events. Only one patient discontinued due to skin discomfort. No serious AEs reported.

Schizophrenia													
1	Andrade, 2013	1	Case report	No information.	Administered by medically qualified family member; no information on monitoring.	tDCS	Anode-F3; Cathode- midway between T3 and P3	3	30	Once to twice daily over a period of 3 years	—	Reduction in frequency and the clinical impact of hallucinations. Clinical effects were improved when increasing sessions from once to twice daily.	No apparent AEs were reported.
2.	Schwippel et al., 2017	1	Case report	Supervised training over multiple sessions.	Self-administered tDCS; no information on home-based supervision.	tDCS	Anode-F4; Cathode- midway between T3 and P3	2	20	3 sessions per week for first 6 months and then once per day onwards for 1 year (n = 400)	—	Patient experienced reliable relief during each tDCS session as a beneficial interruption of the continuous disturbances caused by the hallucinations. Significant improvement of quality of life reported.	Long-term tDCS in the patient appeared safe, with no skin elsions under eelctrodes, no EEG-related abnormalities, no abnormalities or signs of brain damage using MRI, and no deterioration in cognitive abilities.
3.	Desousa, 2017 Geriatric patient	1	Case report	Training provided to family members.	Delivered by trained family members; no information on home-based supervision.	tDCS	Anode-F3; Cathode- midway between T3 and P3	1 to 2	20–30	Once per day for 3 months	—	A progressive and substantial reduction of auditory verbal hallucinations was observed over this period (up to 95% improvement).	No SE reported.
4.	Le Bars et al., 2024 Teenager with schizophrenia	1	Case report	Training of patient and parent with a qualified nurse.	Delivered by trained family members. Session monitoring was achieved using a follow-up sheet with a session schedule, systematic adverse effect screening, and a direct contact number	tDCS	Anode-F3; Cathode- midway between T3 and P3	2	20	10 sessions (twice per day for 5 consecutive days)	—	Reduction in Auditory Hallucinations Rating Scale at end of treatment and two-month follow-up. Auditory-verbal hallucinations were shorter and associated with less anxiety or attention disturbance.	No major AEs were reported.
5.	Pathak et al., 2024	1	Case report	On-site training for caregiver by expert tDCS administrators.	Caregiver-administered tDCS, video-call based remote monitoring by trainers/doctors for initial 3 days and sessions scheduled during working hours to ensure immediate availability of remote support if needed for the rest of the days.	tDCS	Anode-F3; Cathode- midway between T3 and P3	2	20	10 days of home-based tDCS	—	—Feasibility of home-based tDCS was supported.	No AEs reported.

Other psychiatric conditions: Depression and anxiety in other disorders													
1.	Mota et al., 2021; Temporal lobe epilepsy	26	RCT	Clinic-based training.	Self-administered tDCS; on-demand remote supervision via social network, video and telephone calls during treatment.	tDCS	Anode-F3; Cathode- F4	2	20	23 sessions (once per day for 5 days/week for 4 weeks and maintenance phase of once per week for 3 weeks) Maintenance stimulation was clinic-based.	Sham tDCS but no information provided on the protocol.	No group differences in depressive and anxiety symptom reduction between active and sham groups.	No difference in reported AEs between groups. Both groups reported moderate or severe AEs such as headache, itching, tingling, neck pain, drowsiness, change in concentration or mood, or scalp redness. One patient in active group dropped out because of burning discomfort and pain on scalp.
2.	Kim et al., 2024: Mild Cognitive Impaiment	37	RCT	On-site training through a checklist.	Self-administered tDCS; remotely monitored via a server at the hospital by the researchers.	tDCS	Anode-F3; Cathode- F4	2	30	31 sessions (first one in clinic and the other 30 sessions once per day over 6 weeks as home-based)	Sham tDCS but no information provided on the protocol.	No group differences in depressive symptoms and cognitive function between active and sham tDCS. Active tDCS decreased delta activation and increased beta activation on EEG.	No information on the reporting on SEs and AEs.
3.	Lee et al., 2022; Bipolar depression	64	RCT	On-site training by researcher with aid of instructions and videos related to use of tDCS.	Self-administered tDCS; remote access to researcher through voice or video calls to resolve issues regarding device use.	tDCS	Anode-F3; Cathode- F4	2	29	Up to 42 sessions (once per day)	After 30 s ramp-.up and 30 s ramp-down, device was turned off.	No group differences in depressive symptoms between active and sham tDCS. Pain score higher in active than sham group.	No difference in frequency of reported AEs between groups. No treatment-emergent affective switch episodes were reported during trial. There were 4 patients reporting suicidal ideation and 1 reporting an episode of aggressive behaviour.
4.	Rezaei et al., 2025 Bipolar depression	44	Open-label trial	Home-based real time guidance on device setup and usage through video call.	Self-administered tDCS: remotely monitored via video call by research team member.	tDCS	Anode-F3; Cathode- F4	2	30	21 sessions (once per day; 5 sessions per week over 3 weeks and 2 sessions per week for additional 3 weeks)	—	Significant improvement in overall quality of life at the end of treatment and remained elevated at 5-month (from baseline) follow-up. Changes were no longer significant after adjusting for depressive symptoms.	No information on the reporting on SEs and AEs.

Other psychiatric conditions: Binge eating disorder													
1.	Flynn et al., 2024	82	RCT	No information on training.	Self-administered tDCS; tele-supervised.	tDCS combined with attention bias modification training (ABMT)	Anode-F4; Cathode- F3	2	20	10 sessions once per day over 2–3 weeks	Same setup but active stimulation only for 60 s at start and at end of session.	The active tDCS plus ABMT, sham tDCS plus ABMT, and ABMT groups all reduced binge-eating episodes, eating disorder symptoms and related psychopathology at 6-week follow-up compared to baseline, relative to waitlist control. Small-to-moderate effect sizes for change scores suggested superior effects of active tDCS paired with ABMT compared to comparators	Incidence of SEs were not different between active tDCS and sham tDCS groups. Patients in active tDCS group reported mild discomfort during stimulation such as headache. Sham tDCS group reported negligible discomfort due to the stimulation.
2.	Elkfury et al., 2025	40	RCT	Training and staff-assistance provided at first stimulation session.	Self-administered tDCS; weekly scheduled online appointment with research staff to assess device’s functioning and 24/7 contact number in case of assistance.	tDCS combined with nutritional counseling therapy (NCT)	Anode-F4; Cathode- F3	2	20	28 sessions (5 sessions per week for 4 weeks and 8 maintenance sessions once per week)	Same setup, but current automatically turned off 20 s after start of stimulation.	The active tDCS only, NCT only, active tDCS plus NCT, and sham tDCS plus NCT all showed reduction in binge eating scale during treatment and follow-up, without any group differences.	No information on the reporting on SEs and AEs.

Other psychiatric conditions: Attention Deficit Hyperactivity Disorder													
1.	Leffa et al., 2022	64	R	Comprehensive training in device use.	Self-administered tDCS; absence remote monitoring.	tDCS	Anode-F4; Cathode- F3	2	30	28 sessions (once per day for 4 weeks)	Same setup, 30-s ramp-up to 2 mA and 30-s ramp-down to 0 mA at the beginning, in the middle, and at the end of each session.	Active group showed significant reductions in inattention symptoms compared to sham over the different assessment points.	No severe or serious AEs were reported. Mild AEs were more common in active group, especially skin redness, headache, and scalp burn. One patient in sham group dropped out due to neck pain and 2 in the active group dropped out due to depressive symptoms and dizziness.
Other psychiatric conditions: Obsessive Compulsive Disorder (OCD)													
1.	Perera et al., 2023	25	RCT	Supervised training by experienced investigator.	Self-administered tDCS; remote supervision and support via video and phone communication.	Individualized tACS at 25 Hz	AFz and Iz	1.5 peak-to-peak	30	48 sessions (Intensive phase: twice per day for 5 days/week for 3 weeks and Consolidation phase: once daily for 3 days/week for 3 weeks)	Same setup, current at 1.5 mA only in first and last 2 min of session.	Active tACS significantly reduced OCD symptoms from baseline to 6 weeks compared to sham. Trend-level effect maintained at 3-month follow-up.	No serious AEs were observed. Eight patients reported minor AEs, such as headache, phosphene perception, tingling and itching beneath electrodes.

Two studies Vogelmann et al. 2025 and Vogelmann and Baskonus, 2025 were not included in the table as the first is a comparative analysis between home-based and clinic-based tDCS and the second was a letter to editor rather than a study. The stimulation duration reported does not include ramp-up and ramp-down durations. The electrode positions are reported following the 10–20 EEG system. tDCS: transcranial direct current stimulation; tACS: transcranial alternating current stimulation; RCT: randomized controlled trials; TAU: treatment-as-usual; BDI-II: Beck Depression Inventory-II; HDRS: Hamilton Depression Rating Scale, 17-item version; MADRS: Montgomery-Åsberg Depression Rating Scale:

### 3.1 Major depressive disorder

With a prevalence of approximately 4.4% of the global population, regardless of culture or living environment, MDD has received the greatest attention for the development of RS-tES interventions in psychiatry, especially in patients with treatment-resistant depression, which represents approximately 30% of the population.

In a first pilot study examining the safety, feasibility, and efficacy of RS-tES in major depression, two groups were compared. One received 20 sessions (n = 14), and the other received 28 sessions (n = 20) of once daily session of tDCS (2 mA, 30 min, F3-anode and F8-cathode). Participants were monitored via video link during the initial phase of the trial and subsequently through the completion of an online treatment diary. Beneficial clinical outcomes were comparable between the two groups and consistent with those observed in tDCS protocols conducted in clinical settings. Although the study required a certain level of manual dexterity and computer literacy, which could be challenging for some patients, protocol adherence was excellent, with a dropout rate of only 6% and 93% of scheduled sessions completed. These findings highlight a promising avenue for the development of self-administered RS-tES in patients with MDD (Alonzo et al., 2019). Although early studies suggested comparable tolerability between at-home and in-clinic tDCS administration in patients with MDD, more recent evidence indicates a higher frequency of adverse events associated with home-based tDCS as compared with in-clinic administration (Kumpf et al., 2023, Vogelmann and Baskonus, 2025, Vogelmann et al., 2025). In particular, the occurrence of several skin lesions led to a premature termination of a RCT (Kumpf et al., 2023), and comparative analyses showed that sessions with skin lesions were associated with higher impedances (Vogelmann and Baskonus, 2025, Vogelmann et al., 2025). In this case, the adverse events occurred in a context of insufficient safety monitoring, as sessions were not remotely supervised in real time. This underlines the need for implementing real-time remote supervision in home-based tDCS.

Beneficial effects of home-based RS-tES delivered by a caregiver or study companion, rather than self-administered by the patient, have been reported in case series. For instance, beneficial effects were observed with tDCS applied using a multi-channel system (2 mA, 30 min, F3 anode, cathodes on FZ, FC5, and FP1, in a six-week trial, for a total 21 sessions) equipped with real-time monitoring to ensure the safety and efficacy of home-based stimulation in five elderly participants with MDD who completed the study (Cappon et al., 2021). Moreover, a recent open-label multicenter study including 35 participants demonstrated that the same individualized multi-channel RS-tDCS optimized by employing computational models of the electric field may improve depressive symptoms when self-administered in a home-based setting (Ruffini et al., 2024).

Additional case series have highlighted the potential clinical benefits of combining self-administered tDCS (2 mA, 30 min, anode F3, cathode F4) with behavioral therapy delivered via a smartphone application. Two studies from different groups of authors proposed to use the *Flow™ Depression app* that offers interactive, avatar-guided therapy sessions addressing key domains such as behavioral activation, sleep hygiene, healthy nutrition, and mindfulness-based meditation. The protocols consisted of an acute phase of daily sessions, five sessions per week during the first two weeks (Protocol 1) or the first three weeks (Protocol 2), followed by a maintenance phase of twice-weekly sessions for four or three weeks, respectively (18 or 21 sessions in total, for a total of six weeks) (Sobral et al., 2022, n = 7 patients; Borrione et al., 2021, n = 5 patients). However, the interest of combining behavioral therapy delivered via a smartphone application with unsupervised self-administered tDCS was not supported by a subsequent RCT involving 210 adults with MDD. In this study, tDCS was administered in 2 mA 30-minute prefrontal sessions for 15 consecutive weekdays (1 mA, 90-second duration for sham) and twice-weekly sessions for three weeks (Borrione et al., 2024). Another interesting RCT involving 40 participants proposed delivering 21 sessions of home-administered tDCS (anode F3/cathode F4, 2 mA, 30 min once per day) supervised through asynchronous daily monitoring by a trained clinician via a remote supervision platform. This approach, in contrast to real-time video monitoring, is designed to allow multiple patients to use their devices simultaneously, offering greater flexibility for both the patients and the clinicians (Koutsomitros et al., 2023).

Finally, in a well-designed double-blind RCT including 87 patients in the active group and 87 in the sham group, Woodham et al. (2025b) showed that real-time, fully remotely supervised, home-based tDCS could be beneficial for patients with MDD with persistent symptoms at earlier stage of resistance, with or without antidepressant treatment (Woodham et al., 2025b). In this study, tDCS consisted of five sessions per week for three weeks, followed by three sessions per week for seven weeks, resulting in a 10-week treatment phase (2 mA, 30 min, anode F3, cathode F4). All study visits were carried out remotely, allowing the authors to monitor participants’ tDCS sessions in real time; a level of supervision that was not implemented in previous studies reporting negative results (e.g., Borrione et al., 2024). These findings were consistent with previous observations from an open-label pilot study conducted by the same group (Woodham et al., 2022) that also reported that long-term follow-up (until six months) demonstrated high and sustained clinical response rates, regardless of continued tDCS device use (Woodham et al., 2025b).

Other tES parameters have also been tested. A fully remote triple-blind RCT evaluated home-based pulsed transcranial alternating current stimulation (tACS, two 20-min sessions daily for four weeks) in 255 adults with MDD (Gehrman et al., 2024). While the intent-to-treat analysis showed no significant difference between groups, significant improvements in the active vs sham group emerged in highly adherent participants, i.e., participants reporting twice-daily use every day in the first two weeks.

### 3.2 Schizophrenia

Schizophrenia is another major psychiatric condition for which tES has been suggested as a potential treatment approach. Schizophrenia is a chronic and severe psychiatric disorder that affects approximately 0.7 to 1% of the world population. Clinically, schizophrenia is characterized by a broad range of symptoms, including positive symptoms such as hallucinations and delusions, and negative symptoms such as apathy, social withdrawal, and affect flattening. Despite the availability of antipsychotic medications, a significant proportion of patients still experience persistent symptoms and functional impairment, highlighting the need for adjunctive non-pharmacological interventions. In this context, several in-clinic RCTs have reported beneficial effects of tDCS on auditory hallucinations (e.g., Brunelin et al., 2012) and negative symptoms of schizophrenia (e.g., Valiengo et al., 2020).

Despite these encouraging findings, the implementation of RS-tES in schizophrenia has progressed slowly. Interestingly, the earliest uses of home-based tES described in psychiatry were in patients with schizophrenia, with the first case reported as early as 2013, several years before similar work in depression. Despite these early attempts, limited research has been conducted on RS-tES in schizophrenia to date, with only a few published case reports and no RCT available.

In the first case, frontotemporal tDCS (3 mA, 30 min, anode F3, cathode T3P3) was delivered at home in a patient with continuous hallucinations refractory to clozapine, following a first period of in-clinic tDCS delivery (Andrade, 2013). tDCS was delivered by a family member of the patient who was medically qualified, over a total period of nearly three years. A significant reduction of the frequency and the clinical impact of hallucinations was observed. These clinical effects were improved when increasing sessions from once to twice daily. When subsequently reduced to once-daily, the therapeutic effects were maintained, and tDCS was continued with the frequency of daily sessions determined by the patient’s day-to-day needs. Interestingly, several protocol adjustments made during this longitudinal follow-up provide valuable insights for home use of tES. For instance, a loss of treatment efficacy was observed when the frequency of sessions was reduced to once in two days, when the family accidentally interchanged anode and cathode, and when a change in the caregiver led to a shift in electrode positioning. These relapse episodes show the importance of regular monitoring and strict adherence to stimulation parameters to ensure treatment reliability and safety.

In two subsequent cases, home-based tDCS treatment was implemented following an initial in-clinic tDCS delivery and thorough supervised training with the clinical staff. In the first case, at-home tDCS was delivered over a period of 1.5 years (400 sessions) using a reverse frontotemporal montage in a patient with multimodal hallucinations (2 mA, 20 min, anode T3P3, cathode F4, Schwippel et al., 2017). tDCS was delivered three times per week for the first six months and then increased to once-daily. During stimulation, the patient reported consistent relief from the distress and distraction associated with hallucinations, although no lasting reduction beyond the stimulation period was achieved. Safety assessments after long-term use, including neurological and neurocognitive evaluation, EEG recording, structural and diffusion-weighted MRI, and the measurement of a serum marker of neuronal damage, revealed no adverse effects. In the second case, a patient with treatment-resistant auditory hallucinations received daily sessions of frontotemporal tDCS (1 to 2 mA, 20 to 30 min, anode F3, cathode T3P3) at home for three months, delivered by trained family members (Desousa, 2017). A progressive and substantial reduction of hallucinations was observed over this period (up to 95% improvement), with no reported adverse effects.

Two more recent cases further illustrate the feasibility, efficacy, and safety of home-based frontotemporal tDCS (2 mA, 20 min, anode F3, cathode T3P3, twice-daily) for reducing auditory hallucinations in patients with schizophrenia (Le Bars et al., 2024, Pathak et al., 2024). In contrast to the earlier cases, these home-based tDCS treatments were shorter, lasting between 5 and 10 days. In the case of Le Bars et al. (2024), tDCS was delivered by the patient’s parents after a training consisting of a specialized consultation with a qualified nurse. Session monitoring was achieved using a follow-up sheet with a session schedule, systematic adverse effect screening, and a direct contact number. In the case of Pathak et al., a more comprehensive supervised training was provided to caregivers with educational videos, mannequin-based electrode placement practice, and in-person supervised sessions. Caregiver competency across five domains was assessed before and after training. Remote monitoring included video calls during the first three days to ensure safety and proper device use, after which caregivers administered sessions independently. Notably, Le Bars et al. (2024) also highlighted potential cost benefits, with home-based delivery under minimal remote supervision estimated at €231.74 compared with €1,153.37 for hospital-based delivery. However, higher levels of supervision, although increasing treatment costs, are also associated with improved safety (Vogelmann and Baskonus, 2025).

Collectively, these cases support the feasibility, safety, and potential clinical benefit of RS-tDCS in schizophrenia. Nevertheless, further randomized controlled trials are needed.

### 3.3 Other psychiatric conditions

#### 3.3.1 Depression and anxiety in other disorders

Home-based tDCS has also been tested to reduce depression and anxiety symptoms in other conditions, such as temporal lobe epilepsy (TLE; Mota et al., 2021) and patients with mild cognitive impairment (Kim et al., 2024). In TLE, 26 adults with depressive symptoms were randomized to receive either active or sham tDCS (2 mA, 20 min, anode F3, cathode F4) for 20 home-based sessions (five days/week for four weeks), followed by weekly in-clinic maintenance sessions for three weeks. Both interventions led to reductions in depressive symptoms, but no significant differences were found between groups.

Some studies have also investigated the clinical relevance of RS-tES in patients with bipolar depression. Although an initial RCT did not observe the superiority of active tDCS over sham stimulation (Lee et al., 2022), an open-label study including 44 participants (21 sessions of home-based tDCS; 2 mA, 30 min, F3 anode/F4 cathode over six weeks) reported that RS-tDCS was associated with significant improvements in quality of life and functioning, which appeared to be closely related to reductions in depressive symptoms (Rezaei et al., 2025).

#### 3.3.2 Binge eating disorder

Two RCTs have reported the use of self-administered home-based bifrontal tDCS (2 mA, 20 min, anode F4, cathode F3) combined with behavioral interventions for binge eating disorder. In the first one, 82 participants with binge eating disorder received 10 tele-supervised sessions of either active tDCS combined with attention bias modification training, sham tDCS with attention bias modification training, attention bias modification training only, or waitlist control (Flynn et al., 2024). All interventions reduced binge eating episodes and eating disorder symptoms relative to waitlist, with the largest effects observed for active tDCS with attention bias modification training. In the second trial, 40 women with binge eating disorder received 28 sessions (20 intensive, five days/week, and eight maintenance, one day/week) of either active tDCS, nutritional counseling therapy (NCT), sham tDCS with NCT, or active tDCS with NCT (Elkfury et al., 2025). All interventions led to reductions in binge eating episodes, with no synergistic effects or significant differences between groups.

#### 3.3.3 Attention Deficit Hyperactivity Disorder (ADHD)

In a RCT investigating the efficacy of home-based tDCS (30 min, 2 mA, four weeks, anode F3, cathode F4) in 64 drug-free adults with ADHD, active tDCS significantly improved attention compared to sham treatment. The study suggests home-based tDCS as a safe, nonpharmacological option for managing ADHD-related inattention (Leffa et al., 2022).

#### 3.3.4 Obsessive Compulsive Disorder (OCD)

Finally, an RCT investigated the effects of self-administered home-based tACS at individualized alpha frequency in 25 patients with OCD (Perera et al., 2023). After initial supervised training, participants administered tACS (1.5  mA, 30 min, electrodes at AFz and Iz) at home with remote support via video and phone. Sessions were delivered during an intensive phase (twice daily, five days/week for three weeks) followed by a consolidation phase (once daily, three days/week for three weeks). Active tACS significantly reduced OCD symptoms compared to sham.

## 4 Home-based tES in neurological disorders

Neurological disorders are today the leading cause of illness and disability across the globe. In 2021, these disorders affected 3.4 billion individuals and caused 11.1 million deaths worldwide (GBD 2021 Nervous System Disorders Collaborators, 2024). The prevalence of these conditions is forecasted to grow exponentially in the forthcoming decades (GBD 2019 Dementia Forecasting Collaborators, 2022). Neurological disorders, including neurotraumatic and neurodegenerative diseases, comprise a broad spectrum of conditions that impact the central or peripheral nervous system. These disorders can manifest through a range of symptoms, including sensory and motor impairments, cognitive deficits, and seizures. The complexity of their causes, the variability in disease manifestation, and their often progressive course create substantial challenges for patients, caregivers, and healthcare professionals. The use of tES, particularly tDCS, displays exciting potential for reducing neurological burden and delaying disease progression (Antal et al., 2022, Brown and Brown, 2022, Cammisuli et al., 2021). tES treatment protocols commontly entail daily stimulation sessions over several weeks, necessitating regular visits to the clinic. In this section, we will delve into the currently available evidence of different home-based tES methods across varied neurological conditions. The evidence of safety and clinical efficacy as well as the stimulation protocols of the studies cited in this section is summarized in Table 2.

**Table 2:** Summary of studies cited in section on Home-based tES in neurological disorders.

#	Author	N	Study design	Training session	Administration/monitoring	tES type	Electrode position	Intensity (mA)	Duration	# of sessions	Sham protocol	Major finding	Side effect (SE)/adverse event (AE)
Cognitive impairments (Vascular dementia, Alzheimer’s disease (AD), Mild cognitive impairment)													
1	André et al., 2016 Mild vascular dementia	21	RCT	No information provided.	No information provided.	tDCS	Anode-F3; Cathode- F4	2	20	4 consecutive sessions once per day	Setup was same, stimulator was turned off after patients felt initial tingling sensation for 8 s.	Active group showed improved visual short-term memory, verbal working memory, and executive control compared to the sham group.	No AEs were reported.
2	Martorella et al., 2023 Alzheimer’s disease related dementia	40	RCT	Training by research team.	Caregiver-administered tDCS; remote supervision.	tDCS	Anode-C3; Cathode- FP2	2	20	5 sessions (once per day for 5 days)	Setup was same, only 30-s ramp-up at the beginning and the end.	Active group reported clinically meaningful moderate reduction in clinical pain intensity compared to sham group.	No SEs or AEs were reported.
3	Park et al., 2024 AD-related dementia	40	RCT	No information provided.	Caregiver-administered tDCS; remote supervision at scheduled times on weekdays.	tDCS	Anode-C3; Cathode- FP2	2	20	5 consecutive sessions once per day (Monday to Friday)	Setup was same, but only 30-s exposure to 2 mA current.	Greater immediate reduction in scratching behavior was noted in the active group compared to sham. In addition, the active group had a significant impact on reducing the severity and frequency of appetite/eating behaviors and the severity of nighttime behaviors such as disrupted sleep-wake cycle, nighttime wakefulness, and daytime sleepiness compared to sham, with differences noted at three-month post-intervention	No information on the reporting on SEs and AEs.
4	Tippett et al., 2024 Frontotemporal dementia	1	Case report	On-site training by experimenter.	Self-administered tDCS	tDCS combined with computerized cognitive training	Anode-F3; Cathode- F4	No information	No information	46 sessions (once per day over 10 weeks)	—	Clinically significant improvement in global cognition on the Mini-Mental State Exam as well as improvements in language tasks such as syntactic comprehension, semantic processing, and word repetition accuracy following treatment.	No AEs were reported.
5	Im et al., 2019 Early-stage AD	18	RCT	Training of caregiver.	Caregiver-administered tDCS; no information on remote supervision, patient logs were checked at follow-ups.	tDCS	Anode-F3; Cathode- F4	2	29	Daily for 6 months (exact number of sessions not specified)	Setup was same, 30 s ramp-up to 2 mA and 30 s ramp-down to 0 mA.	Active tDCS improved global cognition level and language function, but not delayed recall performance, in patients with early-stage AD compared to sham.	No information on the reporting on SEs and AEs.
6	Grønli et al., 2022 AD	8	Open-label trial	Clinic-based training of caregiver and participant.	Caregiver-administered tDCS; home visit by study leader within 4 days after study started and another 2 home visits and 3 phone calls during 4-month period to check for tDCS feasibility and SEs.	tDCS	Anode-T7; Cathode- F4	2	30	≥ 55 (max. 118) sessions (once per day over 4 months)	—	tDCS did not improve global cognition, attention, language ability, verbal memory, and visuospatial function.	None of the participants reported SEs apart from a slight tingling in the area surrounding the electrodes during the 30-min treatment. No participant discontinued stimulation due to SEs.
7	Bréchet et al., 2021 AD-related dementia	2	Case series	Lab-based training of caregiver following a competency checklist.	Caregiver-administered tACS; remote supervision including the possibility for real-time videoconference with research staff.	Multichannel tACS at 40 Hz	Anodes- CP3, C1; Cathodes-T7, C3, P3, P7 Target: left angular gyrus (BA39/40)	4 (total injected current in montage)	20	70 sessions (once per day for 5 days per week for 14 weeks)	—	Both participants exhibited an improvement in the testing completed every 2 weeks as compared with baseline.	Reported SEs such as tingling and burning sensations were mild. Few occurences of headache and one occurrence of difficulty concentrating. No skin lesions were present.
8	Cappon et al., 2023 AD	8	Open-label trial	Three-day on-site training of study companion by trained research staff.	Study companion-administered tACS; remote monitoring of progression during each session via a smart tablet. On demand remote assistance available.	Multichannel tACS at 40 Hz	Anodes- CP3, C1; Cathodes-T7, C3, P3, P7 Target: left angular gyrus (BA39)	Max. 2 mA for each electrode	20	94 to 134 sessions (acute phase: once per day over 14 weeks [min 5 sessions and max 7 sessions per week], hiatus phase of no stimulation for 12 weeks, and maintenance phase: 2–3 sessions per week for 12 weeks.	—	All participants demonstrated memory enhancement at the end of the acute phase compared to baseline, which was maintained after both the hiatus and the maintenance phase.	Mild SEs were reported during 25% of sessions, moderate during 5%, and severe during 1%. No AEs were reported.
9	Altomare et al., 2023 AD	60	RCT (Protocol paper)	Clinic-based training of study companion by experienced study team member during 5 sessions, and skills test.	Study companion-administered tACS; remote monitoring through verification of stimulation codes and through video calls to check setup. Study team member also available 24/7 by phone for eventual concerns or AEs.	tACS at 40 Hz	Pz (precuneus), right deltoid muscle	2	60	Protocol 1: 80 sessions (once per day 5 times per week [Mon-Fri] over 16 weeks) Protocol 2: 40 sessions of sham tDCS over 8 weeks (once per day 5 times per week[Mon-Fri]), followed by 40 sessions of active tDCS over 8 weeks (once per day 5 times per week [Mon-Fri])	Same setup, current will be discontinued 5 s after start of stimulation.	Larger improvement of cognition, entrainment of gamma oscillations, increased functional connectivity, reduction of pathological burden, and increased cholinergic transmission expected in group receiving Protocol 1 compared to Protocol 2.	Frequency and severity of AEs will be assessed.

Stroke													
1	Mortensen et al., 2016 Haemorrhagic stroke with upper-limb motor impairment	15	RCT	No information provided.	No information provided.	tDCS combined with occupational therapy	Anode-ipsilesional C3 or C4; Cathode- contralesional FP2 or FP1	1.5	20	5 consecutive sessions (once per day)	Same setup, 30-s fade in/fade out sequence at the beginning of the session.	Active group improved grip strength, without any difference in the measure for motor activities for daily living compared to the group receiving sham tDCS paired with occupational therapy. The group difference in grip strength was maintained at the one-week follow-up.	Only mild transient AEs, such as itching, tingling, burning sensation, headache and sleepiness were reported. AEs were reported in both groups, but were more prominent in the active group.
2.	Prathum et al., 2022 Post-stroke with lower- and upper-limn motor impairment	24	RCT	Training of participant and caregiver in use of tDCS for home application.	Self −administered tDCS with assistance of caregiver/ researcher; feedback provided by researcher for the first 3 home-based treatment sessions while visiting the participants at their residence.	tDCS following 60 min of home-based exercise	Anode-ipsilesional C3 or C4; Cathode- contralesional C3 or C4	2	20	12 sessions (once per day 3 times a week for 4 weeks)	Same setup, current applied for first 30 s and then automatically stopped.	Greater motor recovery in both upper and lower limbs in active group compared to the sham group at immediate and 1-month follow-ups. Improvements in lower-limb functional tasks and strength (knee and elbow extensors) were seen only in the active group, while no significant differences were found between groups for upper-limb functional tasks. I	Only mild tDCS-related AEs were reported, including tingling, itching, burning sensation, and headache.
3.	Richardson et al., 2023 Stroke-induced aphasia	2	Open-label trial	In-person training of participant in home use.	Self-administered	tDCS combined with computerized cognitive training	Anode-F3; Cathode- F4	2	20	10 sessions (once per day 5 times a week [Mon-Fri] for 2 weeks)	—	More real words and more relevant words in the post-treatment productions compared to pre-treatment.	No response on AE and tolerability questionnaire that prompted response.
4.	Ko et al., 2022 Post-stroke cognitive impairment	26	RCT	Clinic-based training of participant and/or caregiver through instruction and application of the setup.	Self- or caregiver-administered tDCS; remote supervision: first home-based session supervised by research physician, telephone monitoring 3 times per week and	tDCS combined with computerized cognitive training	Anode-F3; Cathode- F4	2	30	20 sessions (once per day 5 times per week over 4 weeks)	Same setup, the stimulator was turned on for only 10 s, during which the current intensity gradually increased and then decreased until it diminished.	A significant improvement in general cognitive function using the Montreal Cognitive Assessment, but not in other cognitive tests, was observed in the active, but not the sham group after the intervention phase, with larger improvements in patients with moderate rather than mild cognitive impairment.	No serious AEs were reported. No incidents due to unskilled tDCS application or inappropriate stimulation.

Other neurological disorders													
1.	Neophytou et al., 2024 Primary progressive aphasia (PPA)	7	RCT (within-subject randomization)	In-person training of caregiver.	Caregiver-administered tDCS; real-time remote supervision via videocalls for all sessions.	tDCS combined with verbal short-term/working memory treatment	Anode-CP3 (left supramarginal gyrus); Cathode- right cheek	2	20	10 sessions (once per day over two weeks)	Same setup; 30 s ramp-up for sham to 2 mA and immediate ramp-down to 0 mA.	Active tDCS paired with the memory treatment showed a significant effect in improving verbal short-term memory abilities and generalization of this effect to other language abilities, namely, spelling (both real words and pseudowords) and learning (retention and delayed recall) compared to sham combined with memory treatment.	No AEs reported in all patients. Only reported SEs were an initial tingling or itching sensation, reported for both conditions.
2.	George et al., 2025 PPA	10	Case series	No information available.	Self-administered tDCS with caregiver support if needed; remotely supervised.	tDCS combined with personalized word-retrieval training	Anode-F7; Cathode- O1	2	30	20 sessions (once per day over 4 weeks) PWRT is 45 min	—	Enhanced naming accuracy on trained items and confrontation naming, pointing towards a potential offset of lexical retrieval decline in PPA.	No serious AE reported. Mild sensations of tingling and warmth at the initiation of the sessions. No session was discontinued due to tolerability issues.
3.	Pilloni et al., 2024 Multiple sclerosis with hand impairment	65	RCT	Training of participant either in-person or remote for device orientation, training, and tolerability testing.	Self-administered tDCS; real-time remote supervision using secure telehealth videocalls.	tDCS combined with manual dexterity training	Anode-C3; Cathode- FP2	2	20	20 sessions (once per day over	Same setup; ramp-up/down period of target 2.0 mA electrical current for the initial and final 60 s	Active tDCS group showed larger enhancements in manual dexterity, sensory function, and multiple sclerosis-related quality of life compared to the sham group.	Tingling was most frequently reported sensation, followed by itching and warmth sensations. No sessions were stopped due to tolerability issues. Participants reported discomfort > 7 on VAS in only 6 sessions.
4.	Madhavan et al., 2025 ALS	14	RCT	Hands-on training of participant and care-giver following checklist to ensure competency in remote tDCS use.	Self-/caregiver-administered tDCS; real-time video monitoring by researcher.	tDCS	Anode- Lower limb motor cortex; Cathode- contralateral supraorbital region	2	20	Protocol 1: 72 sessions (once per day 3 times per week over 24 weeks) Protocol 2, delayed start: 36 sessions of sham tDCS over 12 weeks (once per day 3 times per week), followed by 36 sessions of active tDCS over 12 weeks (once per day 3 times per week)	No information on sham protocol.	The intervention group exhibited a slower decline in disease severity than the delayed-start group.	No serious AEs present in either group. Itching and tingling were most common SEs with no group difference in frequency. No group difference in occurrence of SEs.
5.	Cha et al., 2016 Mal de Débarquement Syndrome (MDS)	23	RCT	Three face-to-face training sessions in presence.	Self-administered tDCS; remote monitoring via webcam session or pictures of cap position. Daily check-in by patient on personalized web links.	tDCS following 5 sessions of 10 Hz rTMS	Anode-F3; Cathode- F4 for right-handed individuals and reversed for left-handed ones.	1	20	20 sessions (once per day 5 times a week for 4 weeks)	Same setup; 60 s real stimulation given at the beginning of the session with a ramp down.	Active group exhibited significant reductions in the degree of rocking perception and anxiety levels following the post-intervention compared to sham tDCS after the rTMS treatment.	SEs were mild and not different between active and sham tDCS. There were no episodes of skin burns.
6.	Cha et al., 2021 MDS	13	Open-label trial	One-to-one online training of participants for three sessions.	Self-administered tDCS; remote monitoring through device and online monitoring. No real-time staff supervision was present after the training sessions.	Alpha tACS	Fronto-occipital montage with 2 electrodes for anti-phase. For in-phase, two electrodes on scalp and return electrode on left arm.	2 (anti-phase) or 4 mA (in-phase)	20	30 to 165 sessions (5 sessions per week for 4 to 31 weeks, followed by a taper phase: steady reduction in number of sessions by one session every week)	—	Seven participants indicated agreement or strong agreement with the statement that the tACS treatment was beneficial in a blinded survey. During the debriefing interview conducted two to nine months after the final stimulation, five participants described their condition as”great”, experiencing no or minimal symptoms; four reported feeling”good”, with moderate symptoms; and four noted no change from their pre-study baseline	Main SEs included headache, itching, tiredness, and tingling mostly at a level of 3 or less out of 10. One report of 10/10 headache always score not higher than 2 for headache in other sessions. Participants also reported metallic taste in mouth, teeth tingling, phosphine and a sense of head pulsing. No SE was severe enough to discontinue stimulation session.
7.	Mota et al., 2021 Temporal lobe epilepsy with depressive symptoms	26	RCT	On-site training of participant in tDCS use.	Self-administered tDCS; real-time monitoring by research team personnel via an internet communication system.	tDCS	Anode-F3; Cathode- F4	2	20	23 sessions (once per day over 4 weeks, followed by maintenance phase: once per week for 3 weeks)	Same setup; 30 s of progressive pacing (15 s 0–2 mA and 15 s 2–0 mA) at the beginning, middle and end of the application	No differences were observed in depressive and anxious symptoms between active and sham groups.	No increase in seizure frequency during the treatment month compared to month prior to treatment. Moderate or severe AE such as itching, tingling, scalp redness, headache, neck pain, drowsiness, or change in mood or concentration was observed.

Chronic pain													
1.	Brietzke et al., 2020 Fibromyalgia	20	RCT	Clinic-based training by qualified clinician.	Self-administered tDCS; remote monitoring via messaging or through WhatsApp to contact the researcher at any time.	tDCS	Anode-F3; Cathode- F4	2	30	60 sessions (once per day 5 times a week for 12 weeks)	Same setup, 15-s ramp-up (0–2 mA), then a 15-s ramp-down until the current intensity was switched off.	Active group reduced pain intensity and analgesic drug use compared to sham.	No significant difference in cumulative occurrence of burning, itchiness, tingling, and redness is not different between the active and sham groups. The cumulative incidence of neck pain, headache, mood swings, and concentration difficulties were higher in sham than active group. All SEs were classified as mild.
2.	Caumo et al., 2022 Fibromyalgia	48	RCT	Clinic-based training of patient in device use.	Self-administered tDCS; remote monitoring through weekly communication with patients by researcher via WhatsApp and in case of doubts or problems with device, can contact researcher by WhatsApp at any time.	tDCS	Anode-F3; Cathode- F4	2	20	20 sessions (once per day 5 days a week over 4 weeks)	Same setup, device was programmed to offer 30 s of stimulation at the beginning, after 10, and after 20 min during the session.	Active tDCS reduced the Pain Catastrophizing Scale total scores by 51.38% compared to 26.96% in sham group, and reduced Profile of Chronic Pain: Screen total scores by 31.43% compared to 19.15% sham group. The active group improved depressive symptoms, sleep quality and increased the heat pain tolerance	Occurrence of tingling, burning, and redness was higher in active compared to sham group. Higher incidence of burning sensation classified as severe in active group. Most SEs were classified as mild, even in participants who discontinued treatment due to a burning sensation.
3.	Caumo et al., 2024 Fibromyalgia	102	RCT	Clinic-based training of patient in device use.	Self-administered tDCS; remote monitoring through weekly communication with patients by researcher via WhatsApp and in case of doubts or problems with device, can contact researcher by WhatsApp at any time.	tDCS	Arm 1: Anode- F3; Cathode- F4 Arm 2: Anode: C3; Cathode- FP2	2	20	20 sessions (once per day 5 days a week over 4 weeks)	Same setup, device was programmed to offer 30 s of stimulation at the beginning, after 10, and after 20 min during the session.	Superior effects of PreCC-tDCS on pain and disability with a large effect size compared to sham tDCS, while it only moderately reduces pain when applied to DLPFC-tDCS.	Significant difference in burning sensation (classified as severe in active tDCS) between active and sham groups. Incidence of most SEs were similar between groups. Most SEs were classified as mild or moderate, even in patients who discontinued treatment due to a burning sensation.
4.	Jornada et al., 2024 Fibromyalgia	102	RCT	Clinic-based training of patient in device use.	Self-administered tDCS; remote monitoring through weekly communication with patients by researcher via WhatsApp and in case of doubts or problems with device, can contact researcher by WhatsApp at any time.	tDCS	Arm 1: Anode- F3; Cathode- F4 Arm 2: Anode: C3; Cathode- FP2	2	20	20 sessions (once per day 5 days a week over 4 weeks)	Same setup, device was programmed to offer 30 s of stimulation at the beginning, after 10, and after 20 min during the session.	DLPFC-tDCS had a greater impact than anodal PreCC-tDCS on food craving and uncontrolled eating, while PreCC-tDCS was more effective at improving the general symptoms related to fibromyalgia.	SEs were generally mild to moderate.
5.	Serrano et al., 2022 Fibromyalgia	36	RCT	Clinic-based training of patient in device use	Self-administered tDCS; first session at home remotely supervised by research team member. If participant had questions or issues with device, they could contact the research team via WhatsApp anytime.	tDCS	Anode- F3; Cathode- F4	2	20	20 sessions (once per day 5 days a week over 4 weeks)	Same setup, device was programmed to offer 30 s of stimulation at the beginning, after 10, and after 20 min during the session.	Active tDCS improved cognitive performance with large effect size at end of treatment. Compared to sham, active group exhibited improved performance in working memory, verbal and phonemic fluency, and quality of life.	No group difference in AEs such as headache, tingling, burning, redness, and itching. Both groups reported mild SEs and no patients discontinued therapy due to uncomfortable SEs.
6.	Ramasawmy et al., 2024 Fibromyalgia	37	RCT	Two training sessions in the clinic for setting up and operating the device.	Self-administered tDCS; remote group supervision via Zoom by trained interventionist.	tDCS combined with mindfulness meditation	Anode: C3; Cathode- FP2	2	19 min 23 s	10 sessions (once per day 5 times a week [Mon-Fri] over 2 weeks)	Same setup, 17 s ramp-up (0–2 mA), 17 s ramp-down to 0.3 mA, 0.3 mA constant current for 19 min 23 s, and 3 s ramp-down at the end of the session.	Active tDCS paired with meditation did not show superior benefits in reducing pain intensity, affective pain level, psychological distress, and negative affect, or in improving quality of life and sleep quality, compared to sham tDCS combined with meditation group.	No group different in SE occurrences between treatment groups. Most commonly reported SE and AE were skin redness beneath electrodes and headache respectively. No participant discontinued stimulation due to AE or SE. No serious AE was reported.
7.	Caumo et al., 2025 Fibromyalgia	112	RCT	Training of participant on how to use the device.	Self-administered tDCS; remote supervision for initial home-based session and weekly contact through WhatsApp. Participants can contact research team for assistance.	tDCS combined with exercise and pain neuroscience education (PNE)	Anode- F3; Cathode- F4	2	20	20 sessions (once per day 5 times a week over 4 weeks)	Same setup, device was programmed to offer 30 s of stimulation at the beginning, after 10, and after 20 min during the session.	Active tDCS on the left DLPFC combined with exercise and PNE reduced pain severity, disability, and pain interference, particularly in participants prone to a placebo response	The most common AE for active tDCS vs sham tDCS were pain in the stimulation area, tingling, and burning. Headache was more frequent in patients in the sham tDCS vs active group, whereas sleepiness and mood changes were more common in the active tDCS vs sham group. Most symptoms were similar across groups and were classified as mild to moderate, even in those who discontinued due to burning
8.	Lee et al., 2025 Older adults with Knee osteoarthritis	120	RCT	Comprehensive training of participants by research staff at baseline.	Self-administered tDCS; remote monitoring in real time via secure video conferencing.	tDCS	Anode- M1; Cathode- SOPFC (hemisphere not specified)	2	20	15 sessions (once per day 5 times a week over 3 weeks)	Same setup; stimulator was only activated for 30 s at the beginning and end of the session.	Active tDCS was more effective in simultaneously improving pain intensity, pain interference, and pain catastrophizing compared to sham.	No significant SEs were reported.
9.	Martorella et al., 2022 Older adults with Knee osteoarthritis	120	RCT	Training of participants by research staff at baseline.	Self-administered tDCS; real-time remote supervision via videoconference.	tDCS	Anode- M1; Cathode- SOPFC (hemisphere not specified)	2	20	15 sessions (once per day over 3 weeks)	Same setup; the stimulator only delivered 2 mA current for 30 s	Active tDCS significantly reduced pain intensity compared to sham tDCS following treatment.	No serious AEs were reported.
10.	Suchting et al., 2020 Older adults with Knee osteoarthritis	19	Open-label	Training of participants by trained research staff at baseline.	Self-administered tDCS; real-time remote supervision via secured videoconference	tDCS	Anode- M1; Cathode- SOPFC (hemisphere not specified)	2	20	10 sessions (once per day 5 times a week [Mon-Fri] over 2 weeks)	—	Nonparametric tests demonstrated significant improvements to QST measurements from baseline to end of treatment for seven out of 11 QST measures, with small to moderate effect sizes.	No information on the reporting on SEs and AEs.
11.	Ahn et al., 2019 Knee osteoarthritis	30	RCT	Training of participants by trained research staff at baseline.	Self-administered tDCS; real-time remote supervision via secured videoconference	tDCS combined with mindfulness meditation	Anode- M1 contralateral to affected knee; Cathode- SOPFC ipsilateral to affected knee	2	20	10 sessions (once per day 5 times a week [Mon-Fri] over 2 weeks)	Same setup; stimulator was only activated for 30 s at the beginning and end of the session.	Active tDCS paired with active meditation reduced clinical pain and clinical symptoms as well as increased pressure pain thresholds and conditioned pain modulation compared to sham tDCS paired with sham mindfulness meditation.	All participants tolerated combined intervention well without experiencing any serious AEs. No participants reported any SEs associated with the treatment (e.g., itching, burning, headache, fatigue, nervousness, dizziness, or difficulty concentrating).
12.	Carvalho et al., 2018 Neuropathic pain	1	Case report	Training of participant and caretaker.	Self-/caretaker-administered tDCS; real-time monitoring by research team personnel via an internet communication system	tDCS	Anode- M1; Cathode- SOPFC	2	20	5 consecutive sessions (once per day)	—	No worsening of symptoms were noted.	Only minor and transient AEs such as scalp burn sensation, tingling, and skin redness were reported.
13.	Pérez-Borrego et al., 2014 Neuropathic pain	1	Case report	Clinic-based training of caregiver.	Caregiver-administered tDCS; real-time remote supervision via videoconference	tDCS	Anodes- C3, C4; cathode- forehead	1.5	20	Weekly home-based tDCS session	—	Good pain control.	No information on the reporting on SEs and AEs.
14.	Garcia-Larrea et al., 2019 Neuropathic pain	12	Case series (sham-controlled and double-blinded)	Clinic-based training of patient for 5 days of sham tDCS.	Self-administered tDCS; real-time monitoring via server by hospital staff.	tDCS	Anode- motor region contralateral to pain (C3/C4 for upper limb pain, C1/C2 for lower limb pain, C5/C6 for facial pain); cathode- FP1/FP2 contralateral to anode	2 mA and reduced to 1.5 mA if it was uncomfortable for patient	20	25 sessions (once per day 5 days a week: 1 week sham followed by 4 weeks of active tDCS)	Same setup; stimulator was only activated for 30 s at the beginning and end of the session.	6 patients experienced a satisfactory improvement based on a combined measure that included pain levels, medication use, and quality of life. Daily pain reports correlated with such combined assessment, and differentiated responders from non-responders without overlap. Clinical improvement in responders could last up to 6 months.	No serious AE were reported. Superficial burning at electrode position occurred in 2 patients, and nausea/headache in 2 others, all of whom wished to continue the stimulation.
15.	O’Neill et al., 2018 Neuropathic pain	24	RCT (double crossover design)	Clinic-based training of participant.	Self-administered tDCS; no adequate information about monitoring.	tDCS	Anodal stimulation: anode- motor hotspot during TMS mapping contralateral to pain; cathode: supraorbital area contralateral to anode. Reversed for cathodal stimulation.	1.4	20	5 consecutive sessions (once per day) for each intervention block, with a min 2-week washout period	Same setup as M1 anodal tDCS; a constant current of 1.4 mA was delivered only for 5 s (30-second ramp on).	No significant changes in overall pain, anxiety, depression, or quality of life measurement between sham vs anodal tDCS, sham vs cathodal tDCS or anodal vs cathodal tDCS.	Tingling and skin redness were reported during both active and sham stimulations. One patient reported a sharp increase in pain after sham stimulation and withdrew from the study. Five patients reported headache following treatment, which lasted for ∼ 2 h and occurred in both active and sham stimulations. Two patients reported an exacerbation of paresthesia experienced in the affected area at 4-week follow-up.
16.	Antal et al., 2025a, Antal et al., 2025b, Antal et al., 2025c Cancer-related pain	450	RCT (protocol paper)	Clinic-based training of patient and/or caregiver by trained study personnel.	Self- or caregiver-administered tES;	tDCS, 10 Hz tACS	tDCS: Anode: C3; Cathode- FP2 tACS: F3, F4	2 (peak-to-peak for tACS)	20	15 consecutive sessions (once per day)	Same setup; Sham tDCS: 15 s direct current ramp-up to 2 mA, 15 s ramp-down to 0.05 mA, 1130 s of 85 Hz sinusoidal current at 0.05 mA, at the beginning and end of the session Sham tACS: 15 s 10 Hz sinusoidal alternating current ramp-up to 2 mA, 15 s ramp-down to 0.05 mA, and 1130 s of 85 Hz sinusoidal current at 0.05 mA, at the beginning and end of the session	Compared to sham, tDCS and tACS will reduce clinical pain and associated symptoms as well as improve quality of life and global functioning. tDCS might have a stronger effect on pain intensity, while tACS can have a stronger effect of decreasing stress, emotional processing of pain and improving quality of life.	Occurrence of any SE or AE will be reported.

Systematic reviews and meta-analysis were excluded from the table. The stimulation duration reported does not include ramp-up and ramp-down durations. The electrode positions are reported following the 10–20 EEG system. tDCS: transcranial direct current stimulation; tACS: transcranial alternating current stimulation; rTMS: repetitive transcranial magnetic stimulation; RCT: randomized controlled trials; PPA: Primary progressive aphasia; ALS: Amyotrophic Lateral Sclerosis; MDS: Mal de Débarquement Syndrome; DLPFC: dorsolateral prefrontal cortex; PreCC: precentral cortex; BA: Brodmann’s Area; M1: primary motor cortex; SOPFC: supraorbital prefrontal cortex; tES: transcranial electrical stimulation.

### 4.1 Cognitive impairments (vascular dementia, Alzheimer’s disease, mild cognitive impairment)

The application of home-based tES in Alzheimer’s disease (AD) and AD-related dementia (ADRD) has been studied. André et al. (2016) tested the therapeutic potential of four 20-minute sessions of 2 mA anodal at-home tDCS applied to the left dorsolateral prefrontal cortex (DLPFC) (F3/F4 montage based on 10–20 EEG system) in 21 patients with mild vascular dementia. Patients who received the active stimulation demonstrated improved visual short-term memory, verbal working memory, and executive control compared to the sham group.

Five 20-minute sessions of 2 mA anodal RS-tDCS targeting the left precentral cortex (PreCC, also corresponding to the primary motor cortex, M1) (C3/FP2 montage) were conducted to evaluate its analgesic (Martorella et al., 2023) and neuropsychiatric (Park et al., 2024) effects in 40 patients with ADRD. Participants in the active group reported a clinically relevant moderate reduction in pain intensity compared to the sham group. Similar observations were noted in the caregiver-rated perceived clinical pain intensity in the patients, but with a larger difference between active and sham groups. However, these group differences were not maintained at the three-month follow-up (Martorella et al., 2023). A significantly greater immediate reduction in scratching behavior was noted in the active tDCS intervention group compared to sham. In addition, the active group had a significant impact on reducing the severity and frequency of appetite/eating behaviors and the severity of nighttime behaviors such as disrupted sleep-wake cycle, nighttime wakefulness, and daytime sleepiness compared to sham, with differences noted at three-month post-intervention (Park et al., 2024).

Given the dose dependency of the net neuroplastic and behavioral effects of tDCS, multiple studies have also tested the potential of prolonged stimulation periods up to six months. A case report in a patient with frontotemporal dementia by Tippett et al. (2024) showed clinically significant improvement in global cognition on the Mini-Mental State Exam as well as improvements in language tasks such as syntactic comprehension, semantic processing, and word repetition accuracy following 46 sessions of anodal tDCS of the left DLPFC (F3/F4 montage) combined with computerized cognitive training.

Daily 30-minute sessions of 2 mA anodal at-home tDCS targeting the left DLPFC (F3/F4 montage) over six months improved global cognition level and language function, but not delayed recall performance in patients with early-stage AD (n = 12) compared to sham (n = 8) (Im et al., 2019). In another study in AD, 55 or more 30-minute sessions of 2 mA anodal home-based tDCS targeting the left temporal lobe (T7/F4 montage, n = 8) over four months was ineffective in improving global cognition, attention, language ability, verbal memory, and visuospatial function at a four-month follow-up (Grønli et al., 2022).

Despite the extensive, growing body of literature on the potential of tACS in cognitive enhancement in both healthy and diseased populations (Biačková et al., 2024, Wischnewski et al., 2023), only few studies have addressed the efficacy of tACS protocols in neurological conditions. A case series, which included two ADRD patients, to assess the feasibility and tolerability of 70 sessions of 40 Hz home-based tACS administered by a caregiver and under remote supervision showed an improvement in memory every two weeks compared to baseline (Bréchet et al., 2021). Cappon et al. (2023) conducted an open-label study including eight patients with AD testing the effects of multi-channel 40 Hz home-based tACS targeting the left angular gyrus. The intervention included an acute phase comprising a daily 20-minute tACS session over 14 weeks (minimum five sessions and maximum seven sessions per week), a hiatus phase of no stimulation for 12 weeks, and a subsequent maintenance phase of 12 weeks with two to three tACS sessions per week. All participants demonstrated memory enhancement at the end of the acute phase compared to baseline, which was maintained after both the hiatus and the maintenance phase. Nevertheless, the lack of control groups impedes the interpretation of the findings as caused by the stimulation protocol. A randomized clinical trial (RCT) is currently being conducted to test the therapeutic and mechanistic effects of 80 60-minute sessions of 40 Hz at-home tACS targeting the precuneus, applied once per day over 16 weeks, compared to 40 Hz tACS over 8 weeks (Altomare et al., 2023).

### 4.2 Stroke

Another debilitating neurological condition is stroke, which is exponentially increasing due to an aging population and a higher number of young people affected in low- and middle-income countries (Katan and Luft, 2018, Tsao et al., 2023). Kocahasan et al. (2025) conducted a scoping review summarizing the feasibility, safety, and preliminary effects of remotely supervised home-based tDCS in post-stroke recovery. Two randomized clinical trials studied the efficacy of at-home tDCS as an adjunct therapy for motor recovery. (Mortensen et al., 2016) applied five consecutive daily 20-minute sessions of 1.5 mA home-based anodal tDCS targeting the ipsilesional PreCC/M1 simultaneously applied with occupational therapy in hemorrhagic stroke patients with upper-limb motor impairment. Patients in the active group (n = 8) exhibited significantly improved grip strength, without any difference in the measure for motor activities for daily living compared to the group receiving sham tDCS paired with occupational therapy (n = 7). The group difference in grip strength was maintained at the one-week follow-up.

Prathum et al. (2022) included patients with both post-stroke lower- and upper-limb motor impairment in a RCT with a matched-pair design. The participants received a one-hour home-based exercise, following either 20 min of 2 mA anodal home-based tDCS targeting the ipsilesional primary upper-limb motor cortex or sham tDCS three times a week for four weeks. The active tDCS group showed significantly greater motor recovery in both upper and lower limbs compared to the sham group at immediate and 1-month follow-ups, based on Fugl-Meyer assessment scores. Improvements in lower-limb functional tasks and strength (knee and elbow extensors) were seen only in the active group, while no significant differences were found between groups for upper-limb functional tasks. Interestingly, ankle dorsiflexor and hip flexor strength increased only in the sham group. While two investigations examined the effects of repeated at-home tDCS in patients with post-stroke aphasia, none of them could comment on the preliminary efficacy of the intervention on improving language abilities when combined with cognitive training and physical exercise (Pilloni et al., 2022) or computerized language treatment (Richardson et al., 2023). Finally, Ko et al. (2022) conducted an RCT combining 20 30-minute sessions of 2 mA at-home anodal RS-tDCS over the left DLPFC (F3/F4 montage) delivered over four weeks and computerized cognitive training to reduce post-stroke cognitive impairment. A significant improvement in general cognitive function using the Montreal Cognitive Assessment, but not in other cognitive tests, was observed in the active, but not the sham group after the intervention phase, with larger improvements in patients with moderate rather than mild cognitive impairment.

### 4.3 Other neurological disorders

Moreover, the preliminary efficacy of home-based tDCS has also been investigated in other disorders such as primary progressive aphasia (PPA), multiple sclerosis, amyotrophic lateral sclerosis (ALS), and TLE.

A single 20-minute session of 2 mA anodal delivered daily for 10 sessions over two weeks via at-home RS-tDCS directed to the left supramarginal gyrus coupled with verbal short-term/working memory treatment in seven patients with PPA showed a significant improvement in short-term/working memory ability as well as in other language abilities such as spelling, retention, and delayed recall (Neophytou et al., 2024). Each participant underwent a randomized crossover of sham and active stimulation. These effects were not observed in the group receiving sham tDCS combined with the memory treatment. Recently, a case series study in eight patients with PPA testing the effects of a single 30-minute daily session (20 sessions) of 2 mA anodal home-based RS-tDCS targeting the left interior frontal gyrus (F7/O1 montage) over four weeks concurrently applied with a 45-minute personalized word retrieval training has shown enhanced naming accuracy on trained items and confrontation naming, pointing towards a potential offset of lexical retrieval decline in PPA (George et al., 2025). Another study tested the effects of home-based RS-tDCS in multiple sclerosis by comparing 20 sessions of 2 mA of daily anodal tDCS over the left PreCC/M1 (C3/FP2) combined with manual dexterity training to sham tDCS paired with the training in 65 patients with hand impairment (Pilloni et al., 2024). The active tDCS group showed larger enhancements in manual dexterity, sensory function, and multiple sclerosis-related quality of life compared to the sham group. Furthermore, Madhavan et al. (2025) compared the therapeutic benefits of 72 20-minute sessions of 2 mA remotely supervised anodal tDCS targeting the left primary motor cortex representing the lower limbs (the stimulation being delivered thrice per week over 24 weeks) to 36 sessions of sham tDCS over 12 weeks, followed by 36 sessions of anodal tDCS with the same parameters over 12 weeks (delayed start) in 14 patients with ALS. The intervention group exhibited a slower decline in disease severity than the delayed-start group, suggesting a potential positive impact of repeated prolonged tDCS on slowing disease progression.

Interestingly, the therapeutic benefits of home-based repeated tES have also been studied in the Mal de Débarquement Syndrome, a rare vestibular disorder involving a false perception of movement and rocking dizziness. In Cha et al. (2016), participants who received twenty 20-minutes sessions of 1 mA anodal tDCS targeting the left DLPFC (F3/F4 montage) following five sessions of 10 Hz rTMS over the left DLPFC exhibited significant reductions in the degree of rocking perception and anxiety levels following the intervention compared to sham tDCS after the rTMS treatment. A more recent study from the same research group evaluated the impact of prolonged home-based tACS in medically refractory Mal de Débarquement Syndrome in a remotely supervised open-label clinical trial (Cha et al., 2021). The stimulation therapy included five sessions of alpha tACS per week for four to 31 weeks, followed by a four-week taper phase (with a steady reduction in the number of sessions by one session every week). Two electrodes were placed in a fronto-occipital montage delivering either in-phase alpha tACS at 4 mA or anti-phase alpha tACS at 2 mA, while a return electrode was placed on the left arm. Out of the 13 participants, seven in the blinded survey indicated agreement or strong agreement with the statement that the tACS treatment was beneficial. During the debriefing interview conducted two to nine months after the final stimulation, five participants described their condition as”great”, experiencing no or minimal symptoms; four reported feeling”good”, with moderate symptoms; and four noted no change from their pre-study baseline. Furthermore, while a stimulation intervention consisting of 20 sessions of bifrontal anodal home-based tDCS at 2 mA (F3/F4 montage) for 20 min daily over four weeks, followed by a maintenance period of three weeks with stimulation in the laboratory once per week, was shown to be feasible and safe in patients with TLE, no significant differences were observed in depressive and anxious symptoms between active and sham groups (Mota et al., 2021).

### 4.4 Chronic pain

Chronic pain is characterized by pain usually lasting more than three months and therefore lacks the acute warning function of physiological nociception (Treede et al., 2015). Chronic pain can be categorized as chronic primary pain, in which the pain is considered a distinct disease entity (such as fibromyalgia), or as chronic secondary pain, where the pain results from an underlying medical condition (such as neuropathic pain) (Treede et al., 2015). While chronic pain frequently involves neurological mechanisms like central sensitization, its multifaceted nature including its diverse etiologies, among which non-neurological causes and psychosocial influences, does not allow for its classification as a neurological disorder (Borsook, 2012). Given the underlying complex mechanisms of chronic pain, its multidimensional impact, and heterogeneity in treatment response, its management remains a clinical challenge, with a heavy financial burden on healthcare systems (Wang and Doan, 2024). The repeated application of anodal tDCS targeting the PreCC or DLPFC has demonstrated therapeutic benefits in terms of pain relief, reduction of psychological and affective impairment, and disease-related disability across different chronic pain conditions, including neuropathic pain and fibromyalgia (Fregni et al., 2021, Lefaucheur et al., 2017, Wen et al., 2022). Home-based tES provides an avenue for improving accessibility of the neurotechnology to patients with higher adherence to prolonged stimulation paradigms, optimally carried out under remote supervision.

In a recent systematic review and meta-analysis, (Antonioni et al., 2024) analyzed nine RCTs including 446 patients with different chronic pain conditions such as fibromyalgia, knee osteoarthritis, chronic headache, neuropathic pain, and chronic pain in ADRD, with studies included until September 2023. In the meta-analysis, it was found that repeated anodal tDCS in a home-based delivery model may lead to large and clinically meaningful improvement in pain intensity at the end of the intervention (standard mean difference −0,95; low certainty), but only minor non-clinically relevant pain relief at short-term follow-up (SMD −0.50; moderate certainty). No studies explored the application of home-based tACS in chronic pain.

The most studied chronic pain disorder in the field of home-based tDCS is fibromyalgia. Fibromyalgia syndrome is a heterogeneous primary pain condition, characterized by persistent and widespread non-inflammatory musculoskeletal chronic pain. Fibromyalgia affects a significant portion of the global population, with a mean estimated prevalence of 2.7%, and is three times more prevalent in women than in men (Marques et al., 2017, Sarzi-Puttini et al., 2020). FM-associated symptoms commonly include sleep disturbances, fatigue, cognitive impairments, and psychological problems, such as depression and anxiety (Wolfe et al., 2010). A subgroup analysis in the review of Antonioni et al. (2024) included four RCTs in fibromyalgia with 170 participants and showed that repeated home-based tDCS may produce large and clinically meaningful improvement in pain intensity (standard mean difference −1.00; low certainty). A group led by Wolnei Caumo in Brazil first showed in a sham-controlled study on 20 patients with fibromyalgia (10 active, 10 sham) that 60 home-based active tDCS sessions were able to significantly decrease pain intensity as well as analgesic drug use (by 55%). Higher brain-derived neurotrophic factor (BDNF) serum levels were predictive (Brietzke et al., 2020). In a second sham-controlled study on 48 patients with fibromyalgia (32 active, 16 sham), 20 home-based tDCS sessions were able to reduce pain catastrophism, again in correlation with BDNF serum level decrease (Caumo et al., 2022). Both studies were based on anodal tDCS protocol delivered over the left DLPFC. In the last two years, two additional RCTs were published by the same group, implementing home-based tDCS in fibromyalgia. Caumo et al. (2024) compared the efficacy of 20 sessions of 2 mA anodal home-based tDCS targeting the left DLPFC (F3/F4 montage) over four weeks to tDCS over the left PreCC (C3/FP2 montage) in relation to sham in 102 patients with fibromyalgia. They found superior effects of PreCC-tDCS on pain and disability with a large effect size compared to sham tDCS, while it only moderately reduces pain when applied to DLPFC-tDCS. PreCC-tDCS was effective in increasing the heat pain threshold and improving the function of the descending pain inhibitory system. A secondary analysis of the study investigated the effects of tDCS on emotional eating (Jornada et al., 2024), which is a common coping mechanism for alleviating pain-related distress in fibromyalgia (Elkfury et al., 2021). Comparing anodal tDCS and sham independent of target, active stimulation significantly reduced uncontrolled eating, emotional eating, food craving, and waist circumference with large effect sizes. Interaction analyses to address the impact of fibromyalgia symptoms in patients with respect to the stimulation area demonstrated that anodal tDCS of the left DLPFC had a greater impact than anodal PreCC-tDCS on food craving and uncontrolled eating, while PreCC-tDCS was more efficacious at improving the general symptoms related to fibromyalgia. A mechanistic sub-analysis of the study (Alves et al., 2024) revealed that DLPFC-tDCS enhanced beta-3 band connectivity between the left insula and bilateral primary somatosensory cortex, which correlated with sleep quality. In contrast, PreCC-tDCS increased delta band coherence between the right insula and left DLPFC, linked to pain catastrophizing. These findings suggest that home-based anodal tDCS modulates neural connections involved in the emotional and attentional aspects of pain, primarily at lower resting-state EEG frequencies. This modulation of neural oscillations may serve as a marker of its effectiveness in alleviating fibromyalgia symptoms. The same group also showed that cognitive performance, such as working memory and verbal fluency, could be improved in patients with fibromyalgia following home-based anodal tDCS sessions over the left DLPFC (Serrano et al., 2022).

Combinations of home-based tDCS with other non-pharmacological interventions have also been implemented with the goal of enhancing therapeutic benefits in fibromyalgia patients. 10 20-minute sessions of anodal home-based tDCS directed to the left PreCC (C3/FP2 montage) paired with mindfulness meditation did not show superior effects in reducing pain intensity, affective pain level, psychological distress, and negative affect, or in improving quality of life and sleep quality, compared to the group receiving sham tDCS coupled with mindfulness meditation in patients trained in mindfulness (Ramasawmy et al., 2024). However, in a more recent study, Caumo et al. (2025) showed that 20 sessions of 2 mA anodal tDCS delivered with a bi-prefrontal montage (anode on the left DLPFC and cathode on the right DLPFC) over four weeks in combination with exercise and educational guidance decreased pain intensity, disability, and interference, especially in patients prone to a placebo response. The therapeutic benefits of the combined intervention lasted up to three months. Along the line of therapy optimization, an ongoing RCT is being conducted, testing the therapeutic and mechanistic effects of one week of “accelerated” anodal home-based tDCS at 2 mA (15 20-minute tDCS sessions, three sessions per day separated by 2–4 h) comparing targeting anodal tDCS targeting the left PreCC (C3/FP2 montage) versus left DLPFC (F3/F4 montage) in patients with fibromyalgia (https://drks.de/search/de/trial/DRKS00036965).

Addressing other chronic pain disorders, a recent RCT (the largest trial in chronic pain to date, with 123 patients) tested the analgesic efficacy of 15 20-minute sessions of 2 mA anodal tDCS targeting the left PreCC (C3/FP2) in older adults with knee osteoarthritis and found that active stimulation was more effective in simultaneously improving pain intensity, pain interference, and pain catastrophizing compared to sham (Lee et al., 2025). The same group, led by Hyochol Ahn previously reported that home-based tDCS sessions could also reduce experimental pain sensitivity in older adults with knee osteoarthritis (Martorella et al., 2022, Suchting et al., 2020). They also reported that 10 sessions of combining 2 mA anodal RS-tDCS over the PreCC contralateral to the affected knee with mindfulness meditation significantly reduced pain intensity and sensitivity and increased conditional pain modulation, compared to the sham intervention of similar duration pairing sham tDCS with sham meditation (Ahn et al., 2019).

In the context of neuropathic pain, following two case reports (Carvalho et al., 2018, Pérez-Borrego et al., 2014), home-based RS-tDCS protocol delivered with the anode over the PreCC has been proposed in a series of 12 patients (20% sham) (Garcia-Larrea et al., 2019). Daily tDCS sessions were performed during five weeks, and 6 out of the 12 patients achieved satisfactory relief. Clinical improvement in responders could last up to six months. In contrast, another study did not show benefit of five consecutive daily sessions of anodal PreCC-tDCS self-administered at home by 24 patients who had been previously treated by rTMS (13 responders) (O’Neill et al., 2018).

Finally, the PAINLESS-TreatCANCERPAIN project, the largest multicenter RCT to date in chronic pain, is currently ongoing, aiming to recruit 450 patients with cancer-related chronic pain and being the first to compare different tES modalities (Antal et al., 2025b). Its goal is to test the preliminary efficacy and underlying mechanisms of 15 20-minute sessions of daily 2 mA anodal home-based tDCS directed to left PreCC (C3/FP2 montage), compared to daily 10 Hz tACS with a bifrontal montage (F3/F4 montage) at a peak-to-peak current intensity of 2 mA in cancer-related pain.

## 5 Safety, ethical, regulatory aspects

### 5.1 Safety and tolerability of home-based tES

Having outlined the general principles of home-based stimulation and its clinical potential, it is essential to address in more detail the safety and tolerability of this approach. Safety considerations are central, mainly when transferring stimulation procedures from controlled laboratory or clinic environments into patients’ homes, where direct supervision is limited. While tES, and in particular tDCS, has an extensive safety record in clinical and research settings, the question is whether this also holds true for home-based applications. In this section, the term ‘side effect’ denotes a recognized causal link to the intervention and describes an outcome that differs from intended or primary effect, while the term ‘adverse event’ refers to any unintended and unfavorable event temporally linked to the procedure, irrespective whether causality is established (Antal et al., 2025a).

Across more than two decades of research, conventional tES has consistently been shown to be safe when applied within established parameters, with no evidence for serious adverse events in large-scale studies encompassing over 18,000 stimulation sessions (Antal et al., 2025a, Antal et al., 2017, Bikson et al., 2016, Lefaucheur et al., 2017). In line with these findings, RS- tDCS has demonstrated a strong safety profile in numerous studies. When administered under proper protocols and oversight, no serious adverse events have been reported across diverse patient populations (Cappon et al., 2021, Woodham et al., 2025b). For example, a recent fully remote randomized trial for depression (Woodham et al., 2025b) observed no device-related serious adverse events and no cases of induced mania or seizures over 10 weeks of active home tDCS. Similarly, a pilot study in older adults with depression (Cappon et al., 2021) found no serious adverse events over 110 home sessions, with all recorded side effects and adverse events being mild and transient. In fact, tDCS is generally considered to have a benign safety profile comparable to clinic-based applications when used as directed (Alonzo et al., 2019).

Importantly, this robust safety record holds across diverse clinical conditions and age groups. Studies involving adults with major depression (Koutsomitros et al., 2023), chronic pain (Ahn et al., 2019), Parkinson’s disease (Dobbs et al., 2018), and Alzheimer’s dementia (Grønli et al., 2022) all report no serious harm from home-administered tDCS. Even in adolescent populations using related techniques, such as tACS, no serious adverse events have been observed under supervised protocols (Latrèche et al., 2024). These findings underscore that, under controlled conditions, home-based tES is consistently safe and well-tolerated across a wide range of scenarios.

#### 5.1.1 Typical side effects and adverse events

When appropriate devices and protocols are followed (e.g. RS-tDCS), home-based tES is associated with only mild, transient side effects/adverse events. The most common sensations are localized and self-limited, including skin tingling or itching under the electrodes, a slight sensation of burning or warmth, and temporary redness of the scalp at the stimulation sites (Palm et al., 2018). Occasionally, participants report a mild tension headache or fatigue during or after sessions, but these effects occur at comparable rates in sham conditions, generally resolve quickly, and do not require intervention. Crucially, the incidence and intensity of these effects are low. For instance, in a large home RS-tDCS trial for depression (Alonzo et al., 2019; 1,149 sessions), the most frequent effects were a transient burning sensation (reported in ∼ 45% of sessions), tingling (∼22%), electrode-site redness (∼23%), and itching (∼19%) – only ∼ 2% of sessions had any side effect rated as “severe” in intensity (none of which caused lasting harm). Only one out of 1,149 sessions had to be aborted due to discomfort (a brief painful sensation likely from poor electrode contact), and the participant was able to complete all other sessions without incident. Across studies, such as those in depression, pain, or neurological disorders, all reported tDCS side effects and adverse events have been transient, typically ending as soon as stimulation stops. Rare cases of superficial skin burning at scalp electrode position (Garcia-Larrea et al., 2019, Kumpf et al., 2023) have been attributed to approaches not following best-practices (Pilloni et al., 2021, Simani et al., 2025) and burns have not occurred in RS-tDCS.

Importantly, no long-term or cumulative negative effects have been observed in supervised home-based tDCS trials. There are no indications of neurotoxicity or neurologic injury under standard use: for example, participants undergoing daily tDCS for months have shown no adverse changes on cognitive tests or brain imaging attributable to stimulation (Le et al., 2022, Palm et al., 2018). Furthermore, serious complications like seizures have not been triggered by tDCS in home studies; even in a trial targeting patients with epilepsy, active tDCS did not increase seizure frequency relative to sham (Mota et al., 2021). Similarly, no cases of treatment-emergent mania have been observed during home tDCS for depression in unipolar patients (Woodham et al., 2025b), and careful protocols can exclude or monitor those at risk (e.g., bipolar disorder patients) to mitigate this concern. Overall, the evidence supports that home-based tDCS, when appropriate equipment and protocols are incorporated, has a side-effect profile limited to minor, transient sensations, with a risk profile comparable to clinic-based tDCS when best practices are followed.

#### 5.1.2 Tolerability, adherence, and acceptability

Tolerability of home-administered tDCS has generally been excellent, as reflected in high adherence rates and positive patient feedback. Most supervised studies report very low dropout rates and strong completion of sessions. For example, Alonzo et al. (2019) saw a dropout of only 6% over a multi-week home tDCS depression trial, with 93% of all scheduled sessions successfully completed. Other trials similarly achieved near-complete adherence: in Parkinson’s patients, 15 out of 16 participants completed *all* planned sessions (Dobbs et al., 2018); in a recent home-based tDCS study for major depression with asynchronous monitoring, 90% of patients missed three or fewer sessions out of 21, and none dropped out of treatment (Koutsomitros et al., 2023). Such outcomes suggest that patients find at-home tDCS feasible to incorporate into daily life, especially when supported appropriately. Indeed, no patients discontinued these studies due to difficulty with the device or protocol, indicating that the burden of self-administration is low (Le et al., 2022).

Self-reported acceptability is likewise high. Participants typically rate home tDCS sessions as only minimally uncomfortable. Indeed, making tDCS available at home can improve acceptability by eliminating travel and scheduling obstacles, which otherwise often limit treatment uptake (Buchanan et al., 2022). In a sham-controlled trial for binge eating disorder, those receiving real tDCS reported an average discomfort rating of just ∼1.8 out of 10 (sham ∼0.5/10), and importantly, all participants said they would recommend the combined tDCS treatment to others and continue using it in the future (Flynn et al., 2024). Another study of home tDCS for depression found that patients gave very favorable feedback on session tolerability (averaging 4.6 out of 5 stars in post-session ratings), with 91% of all sessions rated as 4- or 5-star experiences (Koutsomitros et al., 2023). Notably, in that study, no patient ever utilized the “pause/stop” safety feature or removed the device mid-session, implying that no session provoked enough discomfort to warrant early termination. This aligns with other reports where participants rarely, if ever, abort sessions due to side effects or adverse events (Cappon et al., 2021).

In summary, across studies home-based tDCS is not only safe but highly tolerable, with patients showing strong compliance and generally positive attitudes toward continuing treatment. However, it should be noted that tolerability depends critically on adequate support. One systematic review (Palm et al., 2018) concluded that studies with insufficient patient training or limited contact had more drop-out or feasibility issues, whereas those providing comprehensive training and frequent check-ins achieved excellent adherence.

#### 5.1.3 Long-term safety and longitudinal tolerability

A key consideration for home-based tDCS is the long-term safety of repeated use, as at-home treatment often involves more sessions than would be feasible in clinic-based settings. Current evidence, though still limited, indicates that extended or maintenance use remains well-tolerated over months or even years, without new safety concerns.

One of the largest datasets comes from a case series in treatment-resistant depression patients who used maintenance home-based tDCS for up to 2.5 years (Le et al., 2022). Across 3,305 recorded sessions, only 8 (0.27%) were associated with adverse events rated as severe, none with lasting consequences. The majority of side effects and adverse events were mild and transient, such as tingling or redness. Neuropsychological testing during follow-up revealed no cognitive decline, consistent with earlier reports that even hundreds of sessions do not affect brain imaging or injury markers (Palm et al., 2018). Only two patients discontinued due to unusual symptoms—one with aggravated pre-existing tinnitus, another with transient blurred vision—both resolving after cessation. Importantly, no patients stopped because of difficulty handling the device, indicating that long-term self-administration is feasible.

Other studies support this benign profile. In Alzheimer’s patients receiving daily tDCS for four months, no troublesome side effects were reported, with only slight tingling noted (Grønli et al., 2022). Caregivers confirmed the absence of adverse changes, and discontinuation occurred in only one participant due to routine fatigue rather than safety issues. Similarly, in an open-label bipolar depression trial (Ghazi-Noori et al., 2024), more than 90% of side effects were mild and their impact decreased over time. Ratings of burden improved from “a bit” affected at baseline to “very much unaffected” at treatment completion. A home-based tDCS study in depression patients also found that the perceived effort of daily sessions lessened over time, with some describing the routine as easier than daily activities (Koutsomitros et al., 2023). These findings suggest that tolerability can even improve with prolonged use.

Overall, available evidence supports the safety and tolerability of home-based tES both in the short and long term when proper safeguards (e.g. RS-tDCS) are applied. The side effect and adverse event spectrum remains confined to mild, transient sensations without evidence of accumulating harm such as cognitive decline or neurological injury (Antal et al., 2025a, Bikson et al., 2016, Palm et al., 2018). Still, regular monitoring is recommended to detect rare or idiosyncratic responses. With such precautions, tDCS appears to be a safe and sustainable neuromodulation tool for home-based treatment paradigms (Paneva et al., 2022).

### 5.2 Ethical and regulatory aspects of home-based tES

Three prominent ethical concerns arising from the home use of tES is 1) safety and risk management, 2) misuse of the technology, and 3) the protection of neurorights. When implemented in accordance with the recommended guidelines (Charvet et al., 2020), respecting the intended use and indication, with suitable training of the participants, patients, and/or their relatives, and with ongoing remote supervision and support from trained clinical or research personnel, home-based tES is a safe and feasible technique, with mostly temporary mild to moderate side effects and adverse events and no reported serious adverse event related to the stimulation to date (please refer to the previous Section).

However, in the case of do-it-yourself (DIY) applications, home users tend to prolong the duration and number of stimulation sessions, even going beyond 100 sessions (Wexler and Reiner, 2018). Although DIY tDCS is generally well-tolerated by home users, the risk of long-term adverse events from large cumulative doses—especially among those who exceed recommended usage with unclear indications—cannot be eschewed (Riggall et al., 2015). Interestingly, as highlighted in the participatory research assessing the stakeholder perspectives on non-invasive brain stimulation conducted by Maier et al. (2024), home users from the DIY community desired tighter oversight of tDCS and regarded the use of unregulated devices from unknown suppliers as unacceptable. They also assessed the risk of side effects, adverse events, and addiction as lower than that associated with medications.

Similar to clinical-based tES, the neuroright to personal identity raises ethical concerns in the context of home-based tES (Antal et al., 2025a), given the neurotechnology’s potential to influence cognition, emotion, and behavior, thereby potentially altering one’s sense of self-identity (Bhidayasiri, 2024). Given that many risks remain hypothetical, it is important to make a distinction between “real and hypothetical problems” before making any regulatory or prohibitive declarations (Bikson and Giordano, 2023). Along the line of Charvet et al. (2020), it is essential to adequately inform home-based tES users about data privacy, where their data will be stored, and about remote monitoring and opt-out processes.

The regulation of tES devices intended for medical use across different continents has been extensively discussed in previous works (Antal et al., 2022, Antal et al., 2024, Antal et al., 2025a, Antal et al., 2025c, Vasquez and Fregni, 2016). Similar to tES devices for clinical use, when it comes to approval of home-tES device in different countries by the national bodies, this rather refers to the specific device and specified use rather than the tES technique and protocol itself. In December 2025, the Flow Neuroscience tDCS device was approved for the treatment of major depressive disorder by the US Food and Drug Administration, making this the first approved home-based tES device in the USA (Bikson et al., 2026). In the latest guidelines (Antal et al., 2025a), the group of experts from the European Society for Brain Stimulation and International Federation for Clinical Neurophysiology recommend the adoption of the RS-tES guidelines (Charvet et al., 2020), which focus on structured staff training, careful participant selection, clear user instruction and consent, standardized procedures for tES protocols and handling adverse events and discontinuation, and routine monitoring, to ensure safety and feasibility of home-based tES. Moreover, we firmly believe that NIBS organizations such as the International Federation of Clinical Neurophysiology (Brain Stimulation Special Interest Group), the International Neuromodulation Society, and the European Society for Brain Stimulation, as well as regulatory bodies like the FDA, MDCG and EMA (European Medicines Agency) can play a leading role in shaping evidence-based frameworks for the safe and ethical deployment of home-based neuromodulation. These standards will be essential for building public trust, ensuring equitable access, and scaling these technologies responsibly.

## 6 Advantages and limitations of home-based NIBS

The advantages and disadvantages are summarized in Table 3.

**Table 3:** Advantages and disadvantages of home-based tES.

**Category**	**Advantage**	**Disadvantage**
Timing and frequency of treatment	Enables higher frequency and regularity, including weekends/holidays, supporting cumulative plasticity. Flexible timing to suit individual chronotypes and daily rhythms (morning vs. evening) to optimize cortical excitability and target effects. Can be paired with daily activities and therapeutic exercises in daily life, enhancing state-dependent plasticity. Self-monitoring and potential AI/wearable guidance for optimal stimulation windows.	Requires self-management; potential mis-timing or inconsistent sessions without clinician oversight.
Long term treatments and repeated daily stimulation sessions	Feasibility of month- to year-long regimens, enabling maintenance therapy and relapse prevention. Supports multiple daily sessions when appropriate, enhancing consolidation and cumulative effects. Fosters autonomy and sustained motivation through self-scheduling and self-administration.	Demands ongoing motivation and adherence; risk of treatment fatigue or burnout without regular clinician support.
Cost/Benefit	Significant reduction in per-session healthcare costs and avoidance of repeated clinic visits. Improves access, reduces travel/time burdens, and enhances health equity. Highly scalable and compatible with telemedicine, potentially lowering system-level costs over time.	High initial purchase cost per user; need for additional devices (headsets, wearables, tablets) and ongoing maintenance. Remote monitoring features add upfront and ongoing costs; economic viability depends on long-term adherence and outcomes.
Compliance	Convenience boosts adherence, with patients able to complete full courses in familiar settings. Promotes autonomy, engagement, and sense of control over treatment. Reduces caregiver burden and enables collaborative home-based care; better real-world monitoring via digital logs.	Dependence on patient discipline; technical issues or user errors can undermine adherence and safety without supervision. Lack of real-time medical oversight; risk of incorrect setup, unmanaged adverse events, mistimed sessions, and non-compliance. Increased potential for misuse or misreporting without supervision.

### 6.1 Advantages of “Home-based” NIBS

#### 6.1.1 Timing and frequency of treatment

Home-based non-invasive brain stimulation enables more frequent and regular sessions, including weekends and holidays. In-clinic protocols are often constrained by scheduling, travel, and staff availability, leading to missed or inflexible sessions. Daily home use supports cumulative plasticity by allowing consistent stimulation that promotes long-term synaptic and network-level changes (Charvet et al., 2015, Bréchet et al., 2021).

Home use also allows stimulation at physiologically optimal times of day, aligned with individual chronotype and therapeutic goals. Morning sessions may enhance alertness or antidepressant effects, while evening stimulation can support memory consolidation or relaxation. This flexibility helps integrate treatment into daily life without disrupting work or social activities (Chmiel and Malinowska, 2025).

At-home stimulation can be paired with real-world cognitive or motor tasks, enhancing state-dependent plasticity. Delivering stimulation before or during training—rarely feasible in clinics—supports more durable, task-specific neural adaptations (Au et al., 2016).

Because physiological responses vary with factors such as mood, sleep, and medication, home-based use enables individualized timing. Patients can track how different schedules affect their symptoms, and advanced systems may use wearable sensors or AI to recommend optimal stimulation windows (Krause and Kadosh, 2014).

Finally, avoiding clinic travel reduces stress and fatigue, promoting a more stable baseline brain state and potentially improving the consistency and efficacy of stimulation.

#### 6.1.2 Long term treatments and repeated daily stimulation sessions

Clinic-based stimulation is often limited by cost, logistics, and personnel. Many chronic neurological and psychiatric conditions, however, require prolonged or maintenance therapy. Home-based systems make extended protocols feasible over months or years (Charvet et al., 2015).

They are particularly suited for maintenance and relapse prevention, allowing intermittent booster sessions or tapered schedules to stabilize improvements in mood, motor function, or cognition. Home systems also allow multiple daily sessions when appropriate, supporting spaced stimulation protocols that enhance consolidation and cumulative effects—especially in motor rehabilitation or cognitive enhancement (Knotkova et al., 2019, Palm et al., 2018).

Self-administered long-term treatment fosters autonomy and engagement, improving adherence and motivation. Patients can schedule sessions during periods of peak alertness or symptom fluctuation, increasing therapeutic relevance and reducing dropout (Charvet et al., 2015, Palm et al., 2018).

#### 6.1.3 Cost/Benefit

Home-based NIBS significantly reduces healthcare costs by eliminating the need for clinic visits, specialized equipment, and staff supervision. Devices for home use—especially tES systems—are compact, inexpensive, and require minimal infrastructure. Reduced travel, missed work, and caregiver burden further lower indirect costs (Charvet et al., 2015).

Although each patient requires a dedicated device and, in some cases, additional monitoring tools, these upfront costs are offset by long-term savings, particularly for chronic conditions requiring frequent treatment. As technology scales and competition increases, device costs are expected to decline. Remote updates, telemedicine integration, and modular designs will further improve cost-efficiency (Bikson et al., 2018).

Over time, improved outcomes, reduced hospitalizations, and better adherence may support insurance coverage or reimbursement. Flexible pricing models such as rentals or subscriptions can also increase accessibility.

#### 6.1.4 Compliance

Home-based NIBS improves treatment adherence by removing barriers such as travel, scheduling, and clinic fatigue. The ability to choose session timing and environment increases consistency and supports long-term therapeutic success (Charvet et al., 2015, Palm et al., 2018).

Greater autonomy enhances patient engagement and satisfaction, which are closely linked to clinical outcomes. Home-based systems also reduce caregiver burden by minimizing clinic visits and enabling shared responsibility in a familiar environment (Knotkova et al., 2019).

Daily use allows caregivers to observe real-world changes in mood, behavior, or motor function, providing valuable insights for clinicians. When combined with digital logging and telemedicine, this supports more responsive and coordinated care (Knotkova et al., 2019, Charvet et al., 2020).

### 6.2 Disadvantages of “Home-based” NIBS

#### 6.2.1 Absence of medical control and misuse

Without real‑time remote supervision (e.g., RS‑tDCS), home‑based NIBS lacks direct clinical oversight. In clinics, trained professionals ensure correct device setup, monitor responses, and adjust protocols as needed. At home, users rely on pre‑set instructions, increasing the risk of incorrect electrode placement, technical errors, and unrecognized adverse events such as headache or skin irritation. The absence of clinician observation also limits timely adjustments to intensity, duration, or targeting, reducing therapeutic precision (Antal et al., 2025a).

Home-based use further increases the risk of misuse and non‑compliance, particularly in patients with cognitive or psychiatric vulnerabilities. Users may skip sessions, apply stimulation at inappropriate times, alter settings, or discontinue treatment prematurely. Underreporting of adverse events and unrealistic expectations about treatment effects may also occur, contributing to inconsistent outcomes. These challenges underscore the need for careful patient selection, thorough training, caregiver involvement, and safety features such as session limits and usage tracking to support responsible long‑term use (Charvet et al., 2020, Charvet et al., 2015, Vogelmann and Baskonus, 2025).

#### 6.2.2 Cost of medical device

Unlike clinic-based systems, where one device serves multiple patients, home-based NIBS requires each user to have a dedicated unit. Additional equipment—such as EEG headsets, sensors, positioning guides, or monitoring tools—may be needed to approximate clinical safety standards, increasing initial costs for patients and healthcare systems (Antal et al., 2025a).

To compensate for the lack of in‑person supervision, home systems often incorporate automated safety mechanisms, telehealth connectivity, and cloud‑based monitoring, which further raise costs. Users engaged in combined cognitive or motor rehabilitation may require supplementary devices or software, adding to financial complexity. For patients needing long-term or daily stimulation, maintaining or replacing equipment over time can become a barrier, raising concerns about economic sustainability and equitable access (Charvet et al., 2015, Charvet et al., 2020).

Despite these challenges, costs are expected to decline as production scales, technology advances, and market competition increases—mirroring trends in other consumer medical technologies. Modular designs, standardized components, and remote software updates may further reduce long‑term expenses (Charvet et al., 2020).

Ultimately, the broader economic benefits of home-based NIBS—fewer clinic visits, improved adherence, reduced hospitalizations, and enhanced quality of life—can offset initial investments. As evidence grows, insurance coverage or reimbursement may become more common, and flexible pricing models (e.g., rentals or subscriptions) could improve accessibility. Although the transition to home use requires upfront investment, its long‑term impact on healthcare efficiency and patient autonomy supports its cost‑effectiveness.

## 7 Future directions

Future home-based tES systems are anticipated to incorporate smart algorithms and real-time feedback for individualized stimulation. By integrating data from wearable sensors (e.g., EEG, heart rate variability, or motion tracking), close-loop controls could dynamically adjust stimulation parameters (e.g., intensity, duration, and frequency) to optimize neurophysiological effects.

As with many consumer health technologies, we can anticipate the miniaturization of tES devices, resulting in lighter, ergonomically refined devices resembling everyday accessories, improving comfort, usability, and adherence, particularly in populations with mobility or cognitive challenges.

Next-generation home-based NIBS platforms will likely be deeply integrated into digital health ecosystems, allowing smooth communication with electronic health records, rehabilitation apps, and telemedicine platforms. Patients and clinicians will be able to view progress dashboards, receive automated alerts, and adjust treatment plans collaboratively. aIntegration with other digital therapies—such as cognitive training, mindfulness, or physical rehabilitation programs—will create multi-modal, synergistic interventions, enhancing neuroplasticity and functional outcomes beyond what stimulation alone can achieve. While digital integration will support the home-based delivery of tES, limited technological literacy may present a barrier for certain demographic groups (e.g., older adults), underscoring the need for highly intuitive and simplified user interfaces.

Artificial Intelligence-driven analytics could support longitudinal data interpretation integrating evidence-based decisions as well as early detection of non-response or adverse events and could recommend timely protocol adjustments. Combined with enhanced remote monitoring tools (e.g., camera-based setup validation or biosignal analysis), such improvements will bring remote care closer to the quality and precision of in-clinic supervision—without requiring constant manual input from healthcare professionals.

As these technologies mature and production scales, reduced costs and expanding reimbursement pathways are expected to make home-based tES broadly accessible, positioning it as a scalable, first-line or adjunct treatment for long-term neurorehabilitation and mental health management.

## 8 Conclusion

Supervised and RS home-based tES, particularly tDCS, represents a transformative advance in neurotherapeutics, integrating digital health and smart device capabilities to enable personalized, biomarker-driven interventions for chronic neurological and pain disorders. Remote supervision and digital training protocols ensure rigorous safety standards and high patient adherence, exceeding 95%, while substantially reducing treatment burden and healthcare costs, especially for individuals with limited mobility or those residing far from specialized centers. Robust clinical trial evidence across multiple populations, including fibromyalgia and major depressive disorder, demonstrates that home-based tES yields significant clinical benefits with consistently mild and transient adverse events, confirming its feasibility and therapeutic potential. Growth in this sector is powered by ongoing technological innovation, the integration of AI for data-driven personalization, and established medical guidelines that uphold strong ethical oversight.

To safely scale this technology, global harmonization of training, stimulation parameters, and regulatory models is required, along with support for reimbursement and healthcare integration to ensure equitable access. Indeed, it is important to highlight that one major factor impeding the accessibility of the given neurotechnology to the general public is the lack of treatment reimbursement by most public health insurances, e.g., in Australia or in most of Europe (Brem and Lehto, 2017, Mathews et al., 2023).

In summary, supervised home-based tES stands poised to deliver substantial, evidence-based improvement in global neurological and pain care, provided that innovation, accessibility, and patient safety advance together under international consensus and collaborative regulatory stewardship. Voice-controlled assistants or AI-based rehabilitation coaches, in the future, can provide real-time instructions, encouragement, and adherence support, especially beneficial for individuals with visual or motor impairments. Remote clinician dashboards can integrate data from all these devices, stimulation logs, rehab performance, and physiological metrics, enabling truly personalized, adaptive, and continuous care from a distance. Besides tES, other methods, such as using home-based rTMS, are under development, the first light-weight home-based high-frequency magnetic stimulator is on the market (Qi et al., 2025).

## Funding

No funding has been provided.

## Declaration of competing interest

The authors declare the following financial interests/personal relationships which may be considered as potential competing interests: PR has received honorarium from neurocare (Germany) for a teaching course, and non-financial support from Sooma Medical™ (Finland), neurocare (Germany), and QuantalX Neuroscience (Israel). The City University of New York holds patents on brain stimulation with MB and KD as inventors. KD consults for Ceragem Medical. MB has equity in Soterix Medical Inc. MB consults, provides expert witness support, received grants, assigned inventions, and/or served on the SAB of SafeToddles, Zabara Family Foundation, Boston Scientific, GlaxoSmithKline, Biovisics, Axonics, Mecta, Lumenis, Halo Neuroscience, Wave Neuroscience, Google-X, i-Lumen, Humm, Allergan(Abbvie), Apple, Ybrain, Ceragem, Ceragem Clinical, Remz. AH is partially employed by neuroConn GmbH. AA is the vice president of the European Society for Brain Stimulation, Member-at-Large at the EMEAC–IFCN, serves as a paid consultant at neuroConn, Ilmenau, and is a paid advisor at Electromedical Products International (Pulvinar), USA. AA is a member of the advisory board at PlatoScience and has non-financial support from Sooma Medical™. AO co-founded Neurek SL.
